# The missing role of gray matter in studying brain controllability

**DOI:** 10.1162/netn_a_00174

**Published:** 2021-03-01

**Authors:** Hamidreza Jamalabadi, Agnieszka Zuberer, Vinod Jangir Kumar, Meng Li, Sarah Alizadeh, Ali Moradi Amani, Christian Gaser, Michael Esterman, Martin Walter

**Affiliations:** Department of Psychiatry and Psychotherapy, University of Tübingen, Tübingen, Germany; Department of Psychiatry and Psychotherapy, University of Tübingen, Tübingen, Germany; Boston University School of Medicine, Department of Psychiatry, Boston, MA, USA; Boston Attention and Learning Laboratory, VA Boston Healthcare System, Boston, MA, USA; Department of Psychiatry and Psychotherapy, Jena University Hospital, Jena, Germany; Max Planck Institute for Biological Cybernetics, Tübingen, Germany; Max Planck Institute for Biological Cybernetics, Tübingen, Germany; Department of Psychiatry and Psychotherapy, Jena University Hospital, Jena, Germany; Department of Psychiatry and Psychotherapy, University of Tübingen, Tübingen, Germanys; School of Engineering, RMIT University, Melbourne, Victoria, Australia; Department of Psychiatry and Psychotherapy, Jena University Hospital, Jena, Germany; Department of Neurology, Jena University Hospital, Jena, Germany; Boston University School of Medicine, Department of Psychiatry, Boston, MA, USA; Boston Attention and Learning Laboratory, VA Boston Healthcare System, Boston, MA, USA; National Center for PTSD, VA Boston Healthcare System, Boston, MA, USA; Department of Psychiatry and Psychotherapy, University of Tübingen, Tübingen, Germany; Clinical Affective Neuroimaging Laboratory, Magdeburg, Germany; Leibniz Institute for Neurobiology, Magdeburg, Germany; Max Planck Institute for Biological Cybernetics, Tübingen, Germany; Department of Psychiatry and Psychotherapy, Jena University Hospital, Jena, Germany

**Keywords:** Network control theory, Gray matter, Brain controllability

## Abstract

Brain controllability properties are normally derived from the white matter fiber tracts in which the neural substrate of the actual energy consumption, namely the gray matter, has been widely ignored. Here, we study the relationship between gray matter volume of regions across the whole cortex and their respective control properties derived from the structural architecture of the white matter fiber tracts. The data suggests that the ability of white fiber tracts to exhibit control at specific nodes not only depends on the connection strength of the structural connectome but additionally depends on gray matter volume at the host nodes. Our data indicate that connectivity strength and gray matter volume interact with respect to the brain’s control properties. Disentangling effects of the regional gray matter volume and connectivity strength, we found that frontal and sensory areas play crucial roles in controllability. Together these results suggest that structural and regional properties of the white matter and gray matter provide complementary information in studying the control properties of the intrinsic structural and functional architecture of the brain.

## Author Summary

Network control theory suggests that the functions of large-scale brain circuits can be partially described with respect to the ability of brain regions to steer brain activity to different states. This ability, often quantified in terms of controllability metrics, has normally been derived from the structural architecture of the white matter fiber tracts. However, gray matter as the substrate that engenders much of the neural processes is widely ignored in this context. In the present work, we study the relationship between regional gray matter volume and control properties across the whole cortex and provide evidence that control properties not only depend on the connection strength of the structural connectome but also depend on sufficient gray matter volume at the host nodes.

## INTRODUCTION

Network control theory, as recently applied to white matter (WM) fiber tracts in the human brain, provides a novel mechanistic framework to describe the ease of switching between different dynamical functional brain states, and the regions that best drive these dynamics (Bassett & Sporns, 2017; Medaglia, 2019; Medaglia, Pasqualetti, Hamilton, Thompson-Schill, & Bassett, 2017). This approach has the potential to inform theories of dynamic cognitive processes, clinical neuroscience, neurodegeneration, and brain reserve. Specifically, there is evidence that these global brain state transitions are impaired in clinical populations (Braun et al., 2019; Jeganathan et al., 2018; Kenett, Beaty, & Medaglia, 2018) and that such impairments can be traced back to specific driver nodes (Jeganathan et al., 2018; Kenett, Beaty, et al., 2018; Muldoon et al., 2016; Zoeller et al., 2019). However, thus far, these control properties have been exclusively derived from WM fiber tracts without the consideration of gray matter (GM) properties. Given the importance of GM properties for cognitive functioning and brain health, and the established interrelationships between white and gray matter, it has been suggested that regional gray matter integrity may be a critical contributor and proxy for network and node controllability (Medaglia, Pasqualetti, et al., 2017; Medaglia, Zurn, Sinnott-Armstrong, & Bassett, 2017).

Several lines of research suggest that GM may be essential to understanding brain controllability. First, GM is a proxy for the quantity of neurons and synaptic densities in a particular region (Lüders, Steinmetz, & Jäncke, 2002), and metabolic energy expenditure is primarily realized through the gray matter cell bodies that scaffold white matter tracts (Zhu et al., 2012). In some neurodegenerative disorders, region-specific lesions of GM only partially agree with corresponding lesions in WM (Agosta et al., 2011; Bodini et al., 2009; Douaud et al., 2007; Raine, Lencz, Bihrle, LaCasse, & Colletti, 2000; Villain et al., 2008), suggesting that GM reserve and WM may provide independent additional information with respect to controllability properties of the structural connectome. Taken together, these studies motivate the hypothesis that the controllability properties suggested by the WM should be partially related to or even predicted by GM integrity. Critically, it has been argued that including GM metrics in control theory will extend traditional volumetrics into network neuroscience (Medaglia, Pasqualetti, et al., 2017). Nevertheless, to our knowledge the nature of the interdependence of controllability properties and GM properties has not been addressed empirically yet.

To tackle this issue, we used two independent datasets to investigate whether—and if so, how—control properties extracted from the structural connectome relate to properties of the gray matter, that is, GM volume that engenders other GM metrics such as surface and thickness (Kong et al., 2015; Winkler et al., 2010). Since previous studies have shown that brain controllability can be largely explained by the connectivity strength of the structural connectome, we also considered whether GM volume could explain additional variance in controllability not accounted for by white matter connectivity. Initially, we investigated how WM and GM factors affect brain controllability on a whole-brain level. In a further step, we identified the brain regions for which controllability was most sensitive to GM and/or WM properties. We discuss our findings with respect to their potential relevance to cognitive and clinical neuroscience.

## METHODS AND MATERIALS

### Data Acquisition

The structural and diffusion datasets are from 65 healthy subjects with the age range of 22 to 36 (28 M, mean age 29.2), which were taken from the Human Connectome Project (HCP, principal investigators: David Van Essen and Kamil Ugurbil; 1U54MH091657; Van Essen et al., 2012). While HCP offers more than 1,100 subjects, the data in the present study are limited by the resources necessary for preprocessing. We have tried to lift the potential bias by including an independent dataset (see the Replication Study section).

#### MRI data specification.

Structural images were acquired with the following specification: T1w MPRAGE, TR 2,400 ms, TE 2.14 ms, TI 1,000 ms, flip angle 8 degrees, field of view (FOV) 224 × 224, 256 slices, voxel size 0.7 mm isotropic, bandwidth 210 Hz/Px, IPAT 2, acquisition time 7:40 min.

Diffusion-weighted imaging (DWI) data were acquired by using a spin-echo EPI sequence with TR 5,520 ms, TE 89.5 ms, flip angle 78 degrees, voxel size, 1.25 mm isotropic, 111 slices, multiband factor, 3, echo spacing, 0.78 ms, b-values 1,000, 2,000, and 3,000 s/mm^2^. For details, see Glasser et al. (2013) and Van Essen et al. (2012).

#### Automated anatomical labeling (AAL) mask definitions and native space transformation.

The 3D anatomy atlas of the AAL2 was acquired from the Neurofunctional Imaging Group (http://www.gin.cnrs.fr/en/tools/aal-aal2/; Tzourio-Mazoyer et al., 2002). It contains 120 regions, which include subcortical structures such as thalamus, caudate, putamen, and pallidum. However, it misses the brain stem. The 12-parameter affine transformation (Jenkinson, Bannister, Brady, & Smith, 2002; Jenkinson & Smith, 2001) was computed for each volunteer’s T1 and nondiffusion image and the MNI space standard brain. The resulting transformation matrix was applied to the left and right AAL brain regions to transform them into the native structural and diffusion space.

#### Structural volume analysis.

The tissue-type segmentation employed SPM12 unified segmentation approach. The process resulted in segmented gray, white, and cerebrospinal fluid (CSF) volumes. In the next step, we determined the volume of the brain, gray matter, and under each AAL atlas region for all subjects. The skull-extracted AC-PC aligned native space NIFTI structural scans were obtained from the Human Connectome database. In the next step, the tissue-type segmentation was applied to delineate the gray matter within the brain using the SPM12 unified segmentation approach (Ashburner & Friston, 2005). This segmentation approach employs a generative model that combines nonlinear registration, tissue classification, and bias correction.

#### Preprocessing and diffusion fit.

The obtained HCP diffusion data were reconstructed using a SENSE1 algorithm (Sotiropoulos et al., 2013). The DWI data were corrected for motion and distortion (Andersson, Skare, & Ashburner, 2003; Andersson & Sotiropoulos, 2015, 2016). Furthermore, preprocessing included unringing, denoising, and tensor analysis implemented in MRtrix (Tournier, Calamante, & Connelly, 2012).

The data were reconstructed using the multi-shell multi-tissue constrained spherical deconvolution (Jeurissen, Tournier, Dhollander, Connelly, & Sijbers, 2014). The resulting orientation distribution function (ODF) was registered to the structural space. The initial tractogram was generated using mrtrix-tckgen, resulting in 100 million streamlines within each subject. In the next step, we applied spherical deconvolution informed filtering of tractograms (SIFT) to reduce the streamline count to 10 million. In the final step, the number of streamlines was determined between AAL brain regions to produce a connectome. The analysis steps are documented in more detail at the MRtrix docs (https://mrtrix.readthedocs.io/en/latest/quantitative_structural_connectivity/structural_connectome.html).

### Network Control Framework

Controllability is one of the fundamental concepts in the control theory. The notion of controllability of a dynamical system was first introduced in Kalman (1963). State (output) controllability of a dynamical system is defined as the possibility of driving states (outputs) of the system from an arbitrary initial condition to any desired values in finite time by applying appropriate control signals (Kailath, 1980). The most famous classic method to ensure state controllability of a dynamical system defined by the noise-free linear discrete-time and time-invariant network model says that the system

x(k+1)=Ax(k)+Bu(k) (1)

y(k)=Cx(k)+Du(k) (2)

is full state controllable if and only if the Kalman’s controllability matrix [*B*, *AB*, …, *A*^*n*−1^*B*] has full rank (Kailath, 1980). In the system represented in Equations 1 and 2, ***x*** ∈ ℝ^*n*^ and ***u*** ∈ ℝ^*p*^ are state and input signals, respectively. *A*, *B*, *C*, and *D* are matrices with appropriate dimensions, where *A* and *B* are called state and input matrices, respectively. When applied in the context of brain controllability, ***x*** describes the activity of brain regions. *A* is an adjacency matrix that represents the interactions between brain regions, and its elements are often the strength of the white tracts connecting two areas (see the Statistical Analysis section below for details). The input matrix *B* identifies the control nodes in the brain that may be confined to one or more brain areas, whose activities are denoted by the corresponding elements of ***x***. While the controllability matrix is a valuable metric to study the overall character of a system, it does not directly quantify the potential ability of different nodes of the system to act as driver nodes. To achieve this, a common practice is to use *Tr*(*W*_*k*_), which is the trace of the controllability Gramian *W*_*k*_ = $\sum_{i=0}^{\infty }$ *A*^*i*^*BB*^*T*^(*A*^*T*^)^*i*^ when the system is controlled from node *k*. Referred to as average controllability (AC), this metric is the most commonly used controllability measure in the neuroimaging literature (Gu et al., 2015; Medaglia, 2019); it is a measure of the average energy required for node*k* to steer the brain into all possible output states (see Tang & Bassett, 2018, for a formal definition). In addition to AC that quantifies the ability of the nodes to drive the system into all potential target states, modal controllability (MC) is another commonly used metric; it is a measure of the ability of the nodes to push the system toward difficult-to-reach states. Formally defined as ϕ_*k*_ = $\sum_{j}^{N}$[1 − $\xi_{j}^{2}$(*A*)]$v_{kj}^{2}$, MC is a scaled measure of difficulty of driving the system toward all *N* modes of *A* from node *k* (Pasqualetti, Zampieri, & Bullo, 2014).

### Statistical Analysis

Linear mixed-effects (LME) regression (Baayen, Davidson, & Bates, 2008) allows us to model the interrelationship among multiple variables and has the ability to accommodate various experimental designs, including repeated measurements, subject variability, and grouping structures, in one unified implementation (Boisgontier & Cheval, 2016). In this paper, we model the interrelationship between brain controllability (AC and MC), GM volume, and connectivity strength, for which we built multiple LMEs. In particular, we include regional gray matter (rGM) and total intracranial volume (TIV) in the LMEs.

To predict brain controllability metrics based on structural measures of the brain, we built a linear mixed-effects regression (Baayen et al., 2008) using a stepwise approach, retaining an effect only if there was a significant difference between the log-likelihood ratio of the two models, based on an analysis of variance (ANOVA, *p* < 0.05). Statistical analysis was performed using the lme4 package in R (Bates, Maechler, Bolker, & Walker, 2014). Specifically, two different models were constructed. The first model is defined by controllability (AC/MC) ∼ TIV + Regions + Nodal degree × rGM + (1|participants), where × denotes the interaction and (1—participants) assigns participants as a random intercept with a fixed slope. The aim was to quantify the contribution of regional gray matter and nodal degree in explaining AC after controlling for the regional differences of AC. In a second model, which is defined as AC/MC ∼ TIV + Regions × Nodal degree + Regions × rGM + (1|participants), we investigate the contribution of regional differences of regional gray matter and regional differences of nodal degree onto AC.

In both models, the volume of the entire brain (TIV) was added as a covariate given its inter-relationship with GM volume (Lüders et al., 2002). In these models all the variables are centered around zero within each subject and normalized using z-transformation. Models were built with the lme4 package in R (Bates et al., 2014).

In these models, the elements Aij of the structural connectivity matrix (i.e., A in Equation 1) represent the number of streamlines between regions i and j. To ensure robustness, we keep only 10% of the strongest connections using the Brain Connectivity Toolbox (Rubinov & Sporns, 2010). Within this scheme, ith node degree is estimated by the sum of all elements of A in the ith row. GM volume is estimated from the unified segmentation approach within SPM12 (see the section on structural volume analysis, above, for details).

#### Null models.

To further test our hypothesis, similar to Lee, Rodrigue, Glahn, Bassett, and Frangou (2020), we built random null models by randomizing the structural connectivity matrix (i.e., A in Equation 1) and estimated the interrelationship between controllability, gray matter, and degree distribution as explained in the section on LME formulation and statistical model comparison, above. Specifically, preserving its degree distribution, we randomized matrix A 1,000 times using the Brain Connectivity Toolbox (Rubinov & Sporns, 2010) and compared the beta values of rGM in the randomized networks with those obtained in the original network.

## RESULTS

### Effects of Gray Matter on Brain Controllability

**Figure 1.**
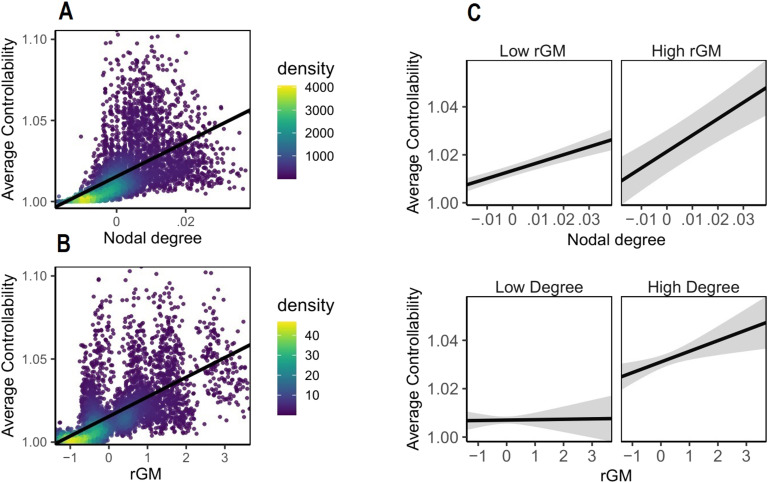
Visualization of interaction effect of nodal degree and rGM in the mixed-effects model predicting average controllability (AC). This effect was controlled for by the TIV and regional differences of average controllability. The figure shows that AC is best explained by WM structure and rGM together. Each dot represents one region from one subject. The density bar shows where the majority of the data are located. (A) Association between nodal degree and AC. (B) Association between rGM and AC. (C) Interaction of rGM and degree on AC, suggesting that the highest levels of AC are reached when both degree and rGM are high together. For visualization, median split was used to classify rGM and degree into high and low. In the original model, both GM and nodal degree were preserved as continuous variables.

In the first step, we investigated whether we could replicate previously reported findings that higher nodal degree relates to higher AC (see Figure 1A). We built a linear mixed-effects model to predict AC based on nodal degree while controlling for TIV and including subjects as a random intercept (for details, see the Supporting Information: Model comparisons, Table C1). Our results, summarized in Figure 1A, replicate previous findings (Gu et al., 2015), suggesting that structural connectivity strength quantified in terms of nodal degree across the whole brain is positively associated with nodal AC. In the second step we investigated whether, beyond this positive association between degree and AC, rGM explains additional variance of AC. To this aim, we extended our model by including GM volume as an additional predictor to nodal degree. Our results show that rGM and nodal degree are both critical to explain AC, and their respective sizes of effects are comparable (β_degree_ = 0.36, *p* value < 0.001; β_rGM_ = 0.44, *p* value < 0.001). Next, we included regions as additional predictors to further explain AC and to improve the fitness of the model. Our results show that rGM and AC are significantly positively associated (see Figure 1B) and interact with nodal degree (β = 0.04, 95% CI [0.01, 0.07], p_bonf_ = 0.01), suggesting that the highest levels of average controllability were best explained with concurrent high rGM and high node degree (see Figure 1C). To verify that the AC cannot not be explained with simpler models, we compared competing models (see the Supporting Information: Model comparisons, Table C1), suggesting that the final model outperformed the competing models. Finally, we used randomized null networks (for details, see the section on null models, above) to investigate whether rGM would remain a significant factor. Our results show that the contribution of rGM in the randomized networks is significantly lower than those in the original networks (*p* value < 0.001). Taken together, our results stress the interdependence of nodal connectivity strength and GM volume for brain controllability.

Finally, in a further step, we used the same model to assess the relationship between MC, rGM, and nodal degree (see the Supporting Information: Modal controllability, Figure B1). Replicating previously reported findings that MC and nodal degree relationships are negatively correlated (see Supporting Information: Modal controllability, Figure B1a), we find that rGM explains a large part of MC variance and that the combination of nodal degree, rGM, and their interaction is necessary.

### Regional Distribution of Average Controllability Based on Gray Matter Volume

Further, we investigated whether this global interdependence of WM and rGM (see the previous section) differs on a regional level. Given our previous results that MC and AC are strongly negatively correlated we focus on AC.

**Figure 2.**
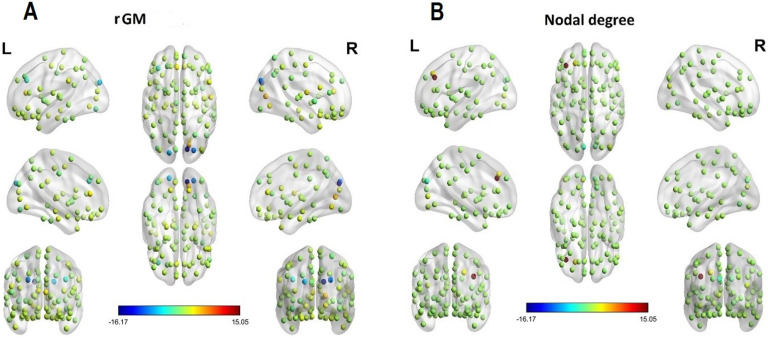
Visualization of interaction effects of mixed-effects model predicting average controllability (AC) based on regional GM (A) and regional nodal degree (B). For visualization, colors represent standardized beta coefficients for effects of rGM and nodal degree, respectively, for each brain region. Higher values indicate a beneficial effect and lower values indicate an impeding effect of rGM/nodal degree on AC.

Our results (see the competing models Supporting Information: Model comparisons, Table C2, and the full outcomes of the winning model in Table S2) show that higher rGM and nodal degree concomitantly are associated with higher AC (see Figure 2; Table S2). Notably, the highest AC levels with higher nodal degree were exhibited in the left frontal middle gyrus (β = 15.11, 95% CI [4.09, 26.13], p_bonf_ = 0.007) and the left superior frontal gyrus (β = 3.01, 95% CI [0.24, 5.78], p_bonf_ = 0.033), which agrees with previous research that also locates driver nodes for AC in the frontal lobes. Further, higher levels of AC were linked to higher levels of nodal degree in the left calcarine (β = 1.78, 95% CI [0.69, 2.86], p_bonf_ = 0.001). There were also regions where higher levels of nodal degree exacerbated AC, with the strongest effects located in the right and left cuneus (right cuneus: β = −1.34, 95% CI [−2.11, −0.57], p_bonf_ = 0.001; left cuneus: β = −2.70, 95% CI [−3.33, −2.08], p_bonf_ < 0.001). When turning to the relation of rGM and AC, higher rGM is associated with higher AC levels in the right calcarine (β = 5.61, 95% CI [4.50, 6.73], p_bonf_ < 0.001), right lingual area (β = 2.98, 95% CI [2.63, 3.33], p_bonf_ < 0.001), and the left and right anterior cingulate (left anterior cingulate: β = 3.76, 95% CI [2.61, 4.91], p_bonf_ < 0.001; right anterior cingulate: β = 2.88, 95% CI [2.28, 3.48], p_bonf_ < 0.001).

There were several regions exhibiting lower AC levels with higher rGM. The strongest effects were found in the right cuneus (β = −16.17, 95% CI [−18.46, −13.88], p_bonf_ < 0.001) and the left frontal middle gyrus (β = −3.34, 95% CI [−6.62, −0.07], p_bonf_ = 0.045). The finding suggests that although on a whole-brain level nodal degree and rGM are concomitantly associated with increased AC, some regions, most notably the left frontal middle gyrus, exhibit higher AC with higher nodal degree, and lower rGM, together (see the Supporting Information, Table S2).

### Replication Study

**Figure 3.**
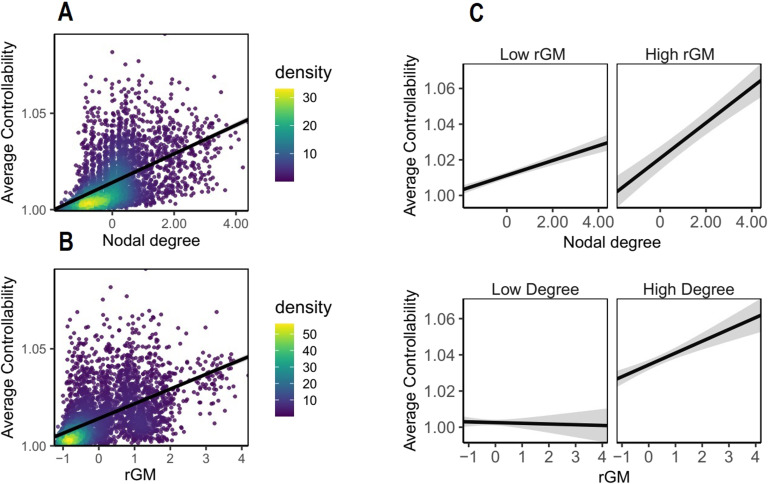
Replication sample. Average controllability (AC) is estimated based on WM structure but also relates to rGM. Each dot represents data from one region of one subject and density bar shows where the majority of data are located. (A) Effect of nodal degree on AC. (B) Effect of rGM on AC. (C) Interaction effect of rGM and nodal degree suggests that the highest levels of AC are reached when both degree and rGM are high together. For visualization, median split was used to classify rGM and degree into high and low. In the original model, both GM and nodal degree were preserved as continuous variables.

To investigate whether the results in the effects of Gray Matter on Brain Controllability section (complementary effects of rGM and nodal degree and AC and MC) are replicable, we used data from a cohort of 48 subjects from another publicly available dataset, where we used a slightly different preprocessing pipeline (for details, see the Supporting Information: Replication study methods). Also in this dataset, nodal degree and rGM increased AC (see Figure 3; for details see Table S3), while the highest AC levels were achieved when both nodal degree and higher rGM were high together (β = 0.08, 95% CI [0.04, 0.12], p_bonf_ = 0.01). Furthermore, rGM and nodal degree both decrease MC, and the lowest values of MC were achieved only for the lowest levels of rGM and nodal degree (see the Supporting Information: Modal controllability, Figure B2). Taken together, these results suggest that this association between rGM and nodal degree is robust and not driven by individual differences in different datasets.

## DISCUSSION

In this work, we investigated how brain volumetrics contribute to global network control properties derived from the structural connectome composed of the white matter fiber tracts. In line with Medaglia, Pasqualetti, et al. (2017), we hypothesized that large-scale network dynamics derived from the structural connectome (here quantified by average and modal brain controllability) would be further explained by structural properties of GM. This work is, to our knowledge, the first attempt to map the interdependence of both metrics, and we discuss our findings with respect to their clinical relevance.

We show that, on average, the amount of rGM directly affects the brain’s availability to dynamically transition between brain states and to adopt new modes of activity. However, levels of brain controllability were best explained when combining information from structural properties of both WM and rGM, suggesting that volumetrics might provide additional information in relating brain controllability to understanding cognition, neurological and neuropsychiatric disorders, and the concept of brain reserve.

### Mediating Role of GM on the Relationship Between WM and Brain Controllability

Our finding that nodal degree is highly predictive of brain controllability agrees with previous work (Gu et al., 2015; Medaglia, 2019), suggesting that the brain’s ability to traverse into easy and difficult-to-reach brain states relies on the strength of structural connectivity, which might reflect the degrees of freedom to steer the transition of brain states. However, our findings suggest that this picture is incomplete. Structural connectivity relies on sufficient support from GM reserves. The highest effects of AC were reached with enhanced nodal degree within frontal regions; this supports the rich literature showing that frontal brain networks play a central role in initiating dynamic reconfigurations during executive cognition. However, increased rGM within that very region was negatively related to brain controllability. While within clinical populations reduced rGM is generally related to neuropathology, there is research suggesting that within healthy subjects, rGM decreases with increases of WM density throughout development from adolescence to adulthood. This finding has been related to reduced quantity of synapses resulting from synaptic pruning (Giorgio et al., 2010), which has been predominantly found in primary visual (calcarine sulcus) and prefrontal cortex (middle frontal gyrus) (Huttenlocher, 1979; Huttenlocher & Dabholkar, 1997). In our data, average brain controllability was maximal when exactly these regions showed reduced rGM and increased connectivity of the white matter connectome. One could speculate that this finding reflects more efficient and developmentally advanced brain functioning in a broad range of tasks potentially related to synchronizing the actions with intentions in a goal-directed way.

### Potential Contribution of Sensory Regions to Brain Controllability

On a functional level, we find several key visual areas that stand out with respect to both average as well as modal controllability. Enhanced rGM in the right cuneus has previously been reported to predict higher error rates in a response inhibition task in bipolar (Haldane, Cunningham, Androutsos, & Frangou, 2008) and has also been related to motor response in functional imaging studies (Booth et al., 2005; Matthews, Simmons, Arce, & Paulus, 2005). We believe that these findings suggest that the function of those primary visual areas goes far beyond unimodal information processing and that primary sensory cortices might occupy more “hub-like” positions in the brain through enhanced long-distance connectivity across brain-wide communities (Esfahlani, Bertolero, Bassett, & Betzel, 2020). Taken together, we speculate that sensory regions could be ideal hot spots for brain controllability nodes. Given their high global interconnectivity, these sensory nodes act potentially as the controllers with respect to the afferent inputs while the other regions act as controllers for efferent demands.

### Linking GM and WM in the Context of Controllability

Cognitive functioning arises from complex reconfigurations across metabolically expensive large-scale networks, facing a trade-off between wiring cost (topological efficiency) and efficient adaptation patterns between multiple neuronal populations (topological value; Bullmore & Sporns, 2012). Recent studies have suggested that the behavioral relevance of this trade-off between topological efficiency and topological value can be described by the brain’s energy expenditure to exhibit control along large-scale structural networks. The ratio of neuronal signaling- to nonsignaling-related metabolic energy expenditure has shown opposite directionalities for white and gray matter (Yu, Herman, Rothman, Agarwal, & Hyder, 2018; Zhu et al., 2012). Here, we speculate that energy expenditure could be one of the key factors linking GM and WM in the framework of controllability analysis. AC relates to the average energy a brain region needs to exert to steer the brain dynamics into all possible brain states (Gu et al., 2015; Kenett, Medaglia, et al., 2018; Liu, Slotine, & Barabasi, 2011) and therefore, more regional gray matter volume is more likely to provide the sufficient energy. In contrast, in absence of sufficient WM tracts, that is, lower nodal degree, rGM cannot fully force the transitions since the energy cannot be exerted. This conception must be expressed on a behavioral level, in that the brain system’s control capacity is especially sensitive to rGM. Indeed, a range of studies have suggested that rGM but not white matter changes relate to abnormal behavioral conditions, such as in antisocial personality disorder (Raine et al., 2000), medication-naive high-functioning children with autism spectrum disorder (Palmen et al., 2005), and alcohol-dependent individuals (Fein et al., 2002). Closely related, MC is strongest when nodal degree and rGM are simultaneously low. MC reflects the ability to drive the brain dynamics toward difficult-to-reach states by changing the modes of activity on the whole-brain level. It is therefore conceivable that similar to the relevance of nodal sparsity to enable optimal MC (Gu et al., 2015), the scarcity of rGM enhances the ability of the host node by targeting only a minimal set of other nodes.

### Limitations: Beyond Linear Full Controllability

Our results in the current study warrant the conclusion that the interplay between gray matter and controllability is of complex nature. Although these results highlight the potential missing role of gray matter in studying brain controllability, there are important aspects that remain yet to be explored. The choice of nonlinear dynamics to define the range of controllability metrics could have considerable effects on our findings. For instance, it is suggested that importance of nodal geometry could actually follow opposite trends when nonlinear and control models are compared (Jiang & Lai, 2019). How the nonlinearity might (re)define the role of rGM for control is an interesting question. Brain controllability metrics considered in the current paper are trajectory unspecific. Studies of dynamical functional and structural connectivity and analysis of structural covariance have reliably shown that brain state trajectories are not random, but rather follow general rules (see Gu et al., 2017; Tang & Bassett, 2018, for recent attempts to accommodate trajectory dependence in the broader context of network control theory). Taken together, we suggest that future work should also include GM in studying brain controllability and further investigate non-linearity of brain controllability.

## ACKNOWLEDGMENTS

HJ was supported by Fortüne grant of Medical Faculty of University of Tübingen (No. 2487-1-0). AZ was supported by the Swiss National Science Foundation (P2ZHP1_181435). MW was supported by EU-ERA-Net: Neuromarket, EU-WIDESPREAD: Fat4BBrain, DFG Wa2673/10, and Neurobiologie motivierten Verhaltens (TPA06) from SFB 779. The authors declare no conflict of interest.

## DATA AVAILABILITY

The data used in the current study are publicly available online. See the Methods section for detail.

## SUPPORTING INFORMATION

Supporting information for this article is available at https://doi.org/10.1162/netn_a_00174.

## AUTHOR CONTRIBUTIONS

Hamidreza Jamalabadi: Conceptualization; Formal analysis; Investigation; Methodology; Project administration; Software; Validation; Writing – original draft; Writing – review & editing. Agnieszka Zuberer: Conceptualization; Formal analysis; Investigation; Methodology; Project administration; Software; Validation; Visualization; Writing – original draft; Writing – review & editing. Vinod Jangir Kumar: Data curation; Writing – review & editing. Meng Li: Data curation; Writing – review & editing. Sarah Alizadeh: Conceptualization; Validation; Writing – review & editing. Ali Moradi Amani: Methodology; Writing – review & editing. Christian Gaser: Investigation; Validation; Writing – review & editing. Michael Esterman: Investigation; Resources; Supervision; Writing – review & editing. Martin Walter: Investigation; Resources; Supervision; Validation; Writing – review & editing.

## FUNDING INFORMATION

Hamidreza Jamalabadi, Fortüne Grant of the Medical Faculty of University of Tübingen, Award ID: 2487-1-0. Agnieszka Zuberer, Swiss National Science Foundation, Award ID: P2ZHP1_ 181435. Martin Walter, ERA-NET NEURON, Award ID: Neuromarket. Martin Walter, EU-WIDESPREAD, Award ID: Fat4BBrain. Martin Walter, DFG, Award ID: Wa2673/10. Martin Walter, Neurobiologie motivierten Verhaltens from SFB 779, Award ID: TPA06.

## TECHNICAL TERMS

Network control theory: A mathematical approach to combine control and network theory. Network control theory can be used to describe dynamics of any system that involves interconnected units, and it allows making predictions about the behavior of the system after perturbation.
Controllability: The property of a dynamical system that quantifies the ability of external inputs to steer the system dynamics toward any desired state.
Average controllability: A controllability metric that quantifies the ability of nodes to steer the network dynamics toward easy-to-reach brain states.
Modal controllability: A controllability metric that quantifies the ability of nodes to steer the network dynamics toward difficult-to-reach states.
Linear mixed-effects model: A form of linear data modeling that allows a hierarchical data structure and dependency between observations by concurrently mapping random and fixed effects.

## References

[bib1] Agosta, F., Pievani, M., Sala, S., Geroldi, C., Galluzzi, S., Frisoni, G. B., & Filippi, M. (2011). White matter damage in Alzheimer disease and its relationship to gray matter atrophy. Radiology, 258(3), 853–863. **DOI:** https://doi.org/10.1148/radiol.10101284, **PMID:** 21177393 21177393 10.1148/radiol.10101284

[bib2] Andersson, J. L., Skare, S., & Ashburner, J. (2003). How to correct susceptibility distortions in spin-echo echo-planar images: Application to diffusion tensor imaging. NeuroImage, 20(2), 870–888. 10.1016/S1053-8119(03)00336-7 14568458

[bib3] Andersson, J. L., & Sotiropoulos, S. N. (2015). Non-parametric representation and prediction of single- and multi-shell diffusion-weighted MRI data using Gaussian processes. NeuroImage, 122, 166–176. **DOI:** https://doi.org/10.1016/j.neuroimage.2015.07.067, **PMID:** 26236030, **PMCID:** PMC4627362 26236030 10.1016/j.neuroimage.2015.07.067 PMC4627362

[bib4] Andersson, J. L., & Sotiropoulos, S. N. (2016). An integrated approach to correction for off-resonance effects and subject movement in diffusion MR imaging. NeuroImage, 125, 1063–1078. **DOI:** https://doi.org/10.1016/j.neuroimage.2015.10.019, **PMID:** 26481672, **PMCID:** PMC4692656 26481672 10.1016/j.neuroimage.2015.10.019 PMC4692656

[bib5] Ashburner, J., & Friston, K. J. (2005). Unified segmentation. NeuroImage, 26(3), 839–851. **DOI:** https://doi.org/10.1016/j.neuroimage.2005.02.018, **PMID:** 15955494 15955494 10.1016/j.neuroimage.2005.02.018

[bib6] Baayen, R. H., Davidson, D. J., & Bates, D. M. (2008). Mixed-effects modeling with crossed random effects for subjects and items. Journal of Memory and Language, 59(4), 390–412. 10.1016/j.jml.2007.12.005

[bib7] Bassett, D. S., & Sporns, O. (2017). Network neuroscience. Nature Neuroscience, 20(3), 353. **DOI:** https://doi.org/10.1038/nn.4502, **PMID:** 28230844, **PMCID:** PMC5485642 28230844 10.1038/nn.4502 PMC5485642

[bib8] Bates, D., Maechler, M., Bolker, B., & Walker, S. (2014). lme4: Linear mixed-effects models using Eigen and S4. R package version, 1(7), 1–23. 10.18637/jss.v067.i01

[bib9] Bodini, B., Khaleeli, Z., Cercignani, M., Miller, D. H., Thompson, A. J., & Ciccarelli, O. (2009). Exploring the relationship between white matter and gray matter damage in early primary progressive multiple sclerosis: An in vivo study with TBSS and VBM. Human Brain Mapping, 30(9), 2852–2861. **DOI:** https://doi.org/10.1002/hbm.20713, **PMID:** 19172648, **PMCID:** PMC6871131 19172648 10.1002/hbm.20713 PMC6871131

[bib10] Boisgontier, M. P., & Cheval, B. (2016). The anova to mixed model transition. Neuroscience & Biobehavioral Reviews, 68, 1004–1005. **DOI:** https://doi.org/10.1016/j.neubiorev.2016.05.034, **PMID:** 27241200 27241200 10.1016/j.neubiorev.2016.05.034

[bib11] Booth, J. R., Burman, D. D., Meyer, J. R., Lei, Z., Trommer, B. L., Davenport, N. D., … Marsel Mesulam, M. (2005). Larger deficits in brain networks for response inhibition than for visual selective attention in attention deficit hyperactivity disorder (ADHD). Journal of Child Psychology and Psychiatry, 46(1), 94–111. **DOI:** https://doi.org/10.1111/j.1469-7610.2004.00337.x, **PMID:** 15660647 15660647 10.1111/j.1469-7610.2004.00337.x

[bib12] Braun, U., Harneit, A., Pergola, G., Menara, T., Schaefer, A., Betzel, R. F., … Chen, J. (2019). Brain state stability during working memory is explained by network control theory, modulated by dopamine D1/D2 receptor function, and diminished in schizophrenia. arXiv:1906.09290. 10.1101/679670

[bib13] Bullmore, E., & Sporns, O. (2012). The economy of brain network organization. Nature Reviews Neuroscience, 13(5), 336. **DOI:** https://doi.org/10.1038/nrn3214, **PMID:** 22498897 22498897 10.1038/nrn3214

[bib14] Douaud, G., Smith, S., Jenkinson, M., Behrens, T., Johansen-Berg, H., Vickers, J., … Matthews, P. M. (2007). Anatomically related grey and white matter abnormalities in adolescent-onset schizophrenia. Brain, 130(9), 2375–2386. **DOI:** https://doi.org/10.1093/brain/awm184, **PMID:** 17698497 17698497 10.1093/brain/awm184

[bib15] Esfahlani, F. Z., Bertolero, M. A., Bassett, D. S., & Betzel, R. F. (2020). Space-independent community and hub structure of functional brain networks. NeuroImage, 116612. **DOI:** https://doi.org/10.1016/j.neuroimage.2020.116612, **PMID:** 32061801 10.1016/j.neuroimage.2020.116612 PMC7104557 32061801

[bib16] Fein, G., Di Sclafani, V., Cardenas, V., Goldmann, H., Tolou-Shams, M., & Meyerhoff, D. J. (2002). Cortical gray matter loss in treatment-naive alcohol dependent individuals. Alcoholism: Clinical and Experimental Research, 26(4), 558–564. **DOI:** https://doi.org/10.1097/00000374-200204000-00017, https://doi.org/101111/j.1530-0277.2002.tb02574.x, **PMID:** 11981133 PMC2435064 11981133

[bib17] Giorgio, A., Watkins, K. E., Chadwick, M., James, S., Winmill, L., Douaud, G., … Johansen-Berg, H. (2010). Longitudinal changes in grey and white matter during adolescence. NeuroImage, 49(1), 94–103. **DOI:** https://doi.org/10.1016/j.neuroimage.2009.08.003, **PMID:** 19679191 19679191 10.1016/j.neuroimage.2009.08.003

[bib18] Glasser, M. F., Sotiropoulos, S. N., Wilson, J. A., Coalson, T. S., Fischl, B., Andersson, J. L., … Polimeni, J. R. (2013). The minimal preprocessing pipelines for the Human Connectome Project. NeuroImage, 80, 105–124. **DOI:** https://doi.org/j.neuroimage.2013.04.127, **PMID:** 23668970, **PMCID:** PMC3720813 23668970 10.1016/j.neuroimage.2013.04.127 PMC3720813

[bib19] Gu, S., Betzel, R. F., Mattar, M. G., Cieslak, M., Delio, P. R., Grafton, S. T., … Bassett, D. S. (2017). Optimal trajectories of brain state transitions. NeuroImage, 148, 305–317. **DOI:** https://doi.org/10.1016/j.neuroimage.2017.01.003, **PMID:** 28088484, **PMCID:** PMC5489344 28088484 10.1016/j.neuroimage.2017.01.003 PMC5489344

[bib20] Gu, S., Pasqualetti, F., Cieslak, M., Telesford,Q. K., Yu, A. B., Kahn, A. E., … Bassett, D. S. (2015). Controllability of structural brain networks. Nature Communications, 6, 8414. **DOI:** https://doi.org/10.1038/ncomms9414, **PMID:** 26423222, **PMCID:** PMC4600713 10.1038/ncomms9414 PMC4600713 26423222

[bib21] Haldane, M., Cunningham, G., Androutsos, C., & Frangou, S. (2008). Structural brain correlates of response inhibition in Bipolar Disorder I. Journal of Psychopharmacology, 22(2), 138–143. **DOI:** https://doi.org/10.1177/0269881107082955, **PMID:** 18308812 18308812 10.1177/0269881107082955

[bib22] Huttenlocher, P. R. (1979). Synaptic density in human frontal cortex-developmental changes and effects of aging. Brain Research, 163(2), 195–205. 10.1016/0006-8993(79)90349-4 427544

[bib23] Huttenlocher, P. R., & Dabholkar, A. S. (1997). Regional differences in synaptogenesis in human cerebral cortex. Journal of Comparative Neurology, 387(2), 167–178. 10.1002/(SICI)1096-9861(19971020)387:2<167::AID-CNE1>3.0.CO;2-Z 9336221

[bib24] Jeganathan, J., Perry, A., Bassett, D. S., Roberts, G., Mitchell, P. B., & Breakspear, M. (2018). Fronto-limbic dysconnectivity leads to impaired brain network controllability in young people with bipolar disorder and those at high genetic risk. NeuroImage: Clinical, 19, 71–81 . **DOI:** https://doi.org/10.1016/j.nicl.2018.03.032 , **PMID:** 30035004, **PMCID:** PMC6051310 30035004 10.1016/j.nicl.2018.03.032 PMC6051310

[bib25] Jenkinson, M., Bannister, P., Brady, M., & Smith, S. (2002). Improved optimization for the robust and accurate linear registration and motion correction of brain images. NeuroImage, 17(2), 825–841. **DOI:** https://doi.org/10.1006/nimg.2002.1132, **PMID:** 12377157 12377157 10.1016/s1053-8119(02)91132-8

[bib26] Jenkinson, M., & Smith, S. (2001). A global optimisation method for robust affine registration of brain images. Medical Image Analysis, 5(2), 143–156. 10.1016/S1361-8415(01)00036-6 11516708

[bib27] Jeurissen, B., Tournier, J.-D., Dhollander, T., Connelly, A., & Sijbers, J. (2014). Multi-tissue constrained spherical deconvolution for improved analysis of multi-shell diffusion MRI data. NeuroImage, 103, 411–426. **DOI:** https://doi.org/10.1016/j.neuroimage.2014.07.061, **PMID:** 25109526 25109526 10.1016/j.neuroimage.2014.07.061

[bib28] Jiang, J., & Lai, Y.-C. (2019). Irrelevance of linear controllability to nonlinear dynamical networks. Nature Communications, 10(1), 1–10. **DOI:** https://doi.org/10.1038/s41467-019-11822-5, **PMID:** 31481693, **PMCID:** PMC6722065 10.1038/s41467-019-11822-5 PMC6722065 31481693

[bib29] Kailath,T. (1980). Linear systems (Vol. 156). Prentice-Hall.

[bib30] Kalman, R. E. (1963). Mathematical description of linear dynamical systems. Journal of the Society for Industrial and Applied Mathematics, Series A: Control, 1(2), 152–192. 10.1137/0301010

[bib31] Kenett, Y. N., Beaty, R. E., & Medaglia, J. D. (2018). A computational network control theory analysis of depression symptoms. Personality Neuroscience, 1. **DOI:** https://doi.org/10.1017/pen.2018.15, **PMID:** 30706049, **PMCID:** PMC6349380 10.1017/pen.2018.15 PMC6349380 30706049

[bib32] Kenett, Y. N., Medaglia, J. D., Beaty, R. E., Chen, Q., Betzel, R. F., Thompson-Schill, S. L., & Qiu, J. (2018). Driving the brain towards creativity and intelligence: A network control theory analysis. Neuropsychologia. **DOI:** https://doi.org/10.1016/j.neuropsychologia.2018.01.001, **PMID:** 29307585, **PMCID:** PMC6034981 10.1016/j.neuropsychologia.2018.01.001 PMC6034981 29307585

[bib33] Kong, L., Herold, C. J., Zöllner, F., Salat, D. H., Lässer, M. M., Schmid, L. A., … Schad, L. R. (2015). Comparison of grey matter volume and thickness for analysing cortical changes in chronic schizophrenia: A matter of surface area, grey/white matter intensity contrast, and curvature. Psychiatry Research: Neuroimaging, 231(2), 176–183. **DOI:** https://doi.org/10.1016/j.pscychresns.2014.12.004, **PMID:** 25595222 10.1016/j.pscychresns.2014.12.004 25595222

[bib34] Lee, W. H., Rodrigue, A., Glahn, D. C., Bassett, D. S., & Frangou, S. (2020). Heritability and cognitive relevance of structural brain controllability. Cerebral Cortex, 30(5), 3044–3054. **DOI:** https://doi.org/10.1093/cercor/bhz293, **PMID:** 31838501, **PMCID:** PMC7197079 31838501 10.1093/cercor/bhz293 PMC7197079

[bib35] Liu, Y. Y., Slotine, J. J., & Barabasi, A. L. (2011). Controllability of complex networks. Nature, 473(7346), 167–173. **DOI:** https://doi.org/10.1038/nature10011, **PMID:** 21562557 21562557 10.1038/nature10011

[bib36] Lüders, E., Steinmetz, H., & Jäncke, L. (2002). Brain size and grey matter volume in the healthy human brain. Neuroreport, 13(17), 2371–2374. **DOI:** https://doi.org/10.1097/00001756-200212030-00040, **PMID:** 12488829 12488829

[bib37] Matthews, S. C., Simmons, A. N., Arce, E., & Paulus, M. P. (2005). Dissociation of inhibition from error processing using a parametric inhibitory task during functional magnetic resonance imaging. Neuroreport, 16( 7), 755–760. **DOI:** https://doi.org/10.1097/00001756-200505120-00020, **PMID:** 15858420 15858420 10.1097/00001756-200505120-00020

[bib38] Medaglia, J. D. (2019). Clarifying cognitive control and the controllable connectome. Wiley Interdisciplinary Reviews: Cognitive Science, 10(1), e1471. **DOI:** https://doi.org/10.1002/wcs.1471, **PMID:** 29971940, **PMCID:** PMC6642819 29971940 10.1002/wcs.1471 PMC6642819

[bib39] Medaglia, J. D., Pasqualetti, F., Hamilton, R. H., Thompson-Schill, S. L., & Bassett, D. S. (2017). Brain and cognitive reserve: Translation via network control theory. Neuroscience & Biobehavioral Reviews, 75, 53–64. **DOI:** https://doi.org/10.1016/j.neubiorev.2017.01.016, **PMID:** 28104411, **PMCID:** PMC5359115 28104411 10.1016/j.neubiorev.2017.01.016 PMC5359115

[bib40] Medaglia, J. D., Zurn, P., Sinnott-Armstrong, W., & Bassett, D. S. (2017). Mind control as a guide for the mind. Nature Human Behaviour, 1(6). 10.1038/s41562-017-0119

[bib41] Muldoon, S. F., Pasqualetti, F., Gu, S., Cieslak, M., Grafton, S. T., Vettel, J. M., & Bassett, D. S. (2016). Stimulation-based control of dynamic brain networks. PLoS Computational Biology, 12(9), e1005076. **DOI:** https://doi.org/10.1371/journal.pcbi.1005076, **PMID:** 27611328, **PMCID:** PMC5017638 27611328 10.1371/journal.pcbi.1005076 PMC5017638

[bib42] Palmen, S. J., Pol, H. E. H., Kemner, C., Schnack, H. G., Durston, S., Lahuis, B. E., … Van Engeland, H. (2005). Increased gray-matter volume in medication-naive high-functioning children with autism spectrum disorder. Psychological Medicine, 35(4), 561–570. **DOI:** https://doi.org/10.1017/S0033291704003496, **PMID:** 15856726 15856726 10.1017/s0033291704003496

[bib43] Pasqualetti, F., Zampieri, S., & Bullo, F. (2014). Controllability metrics, limitations and algorithms for complex networks. IEEE Transactions on Control of Network Systems, 1(1), 40–52. 10.1109/TCNS.2014.2310254

[bib44] Raine, A., Lencz, T., Bihrle, S., LaCasse, L., & Colletti, P. (2000). Reduced prefrontal gray matter volume and reduced autonomic activity in antisocial personality disorder. Archives of General Psychiatry, 57(2), 119–127. **DOI:** https://doi.org/10.1001/archpsyc.57.2.119, **PMID:** 10665614 10665614 10.1001/archpsyc.57.2.119

[bib45] Rubinov, M., & Sporns, O. (2010). Complex network measures of brain connectivity: Uses and interpretations. NeuroImage, 52(3), 1059–1069. **DOI:** https://doi.org/10.1016/j.neuroimage.2009.10.003, **PMID:** 19819337 19819337 10.1016/j.neuroimage.2009.10.003

[bib46] Sotiropoulos, S. N., Moeller, S., Jbabdi, S., Xu, J., Andersson, J. L., Auerbach, E. J., … Lenglet, C. (2013). Effects of image reconstruction on fiber orientation mapping from multichannel diffusion MRI: Reducing the noise floor using SENSE. Magnetic Resonance in Medicine, 70(6), 1682–1689. **DOI:** https://doi.org/10.1002/mrm.24623, **PMID:** 23401137, **PMCID:** PMC3657588 23401137 10.1002/mrm.24623 PMC3657588

[bib47] Tang, E., & Bassett, D. S. (2018). Colloquium: Control of dynamics in brain networks. Reviews of Modern Physics, 90(3), 031003. 10.1103/RevModPhys.90.031003

[bib48] Tournier, J. D., Calamante, F., & Connelly, A. (2012). MRtrix: Diffusion tractography in crossing fiber regions. International Journal of Imaging Systems and Technology, 22(1), 53–66. 10.1002/ima.22005

[bib49] Tzourio-Mazoyer, N., Landeau, B., Papathanassiou, D., Crivello, F., Etard, O., Delcroix, N., … Joliot, M. (2002). Automated anatomical labeling of activations in SPM using a macroscopic anatomical parcellation of the MNI MRI single-subject brain. NeuroImage, 15(1), 273–289. **DOI:** https://doi.org/10.1006/nimg.2001.0978, **PMID:** 11771995 11771995 10.1006/nimg.2001.0978

[bib50] Van Essen, D. C., Ugurbil, K., Auerbach, E., Barch, D., Behrens, T., Bucholz, R., … Curtiss, S. W. (2012). The Human Connectome Project: A data acquisition perspective. NeuroImage, 62(4), 2222–2231. **DOI:** https://doi.org/10.1016/j.neuroimage.2012.02.018, **PMID:** 22366334, **PMCID:** PMC3606888 22366334 10.1016/j.neuroimage.2012.02.018 PMC3606888

[bib51] Villain, N., Desgranges, B., Viader, F., De La Sayette, V., Mézenge, F., Landeau, B., … Chételat, G. (2008). Relationships between hippocampal atrophy, white matter disruption, and gray matter hypometabolism in Alzheimer’s disease. Journal of Neuroscience, 28(24), 6174–6181. **DOI:** https://doi.org/10.1523/JNEUROSCI.1392-08.2008, **PMID:** 18550759, **PMCID:** PMC2902815 18550759 10.1523/JNEUROSCI.1392-08.2008 PMC2902815

[bib52] Winkler, A. M., Kochunov, P., Blangero, J., Almasy, L., Zilles, K., Fox, P. T., … Glahn, D. C. (2010). Cortical thickness or grey matter volume? The importance of selecting the phenotype for imaging genetics studies. NeuroImage, 53(3), 1135–1146. **DOI:** https://doi.org/10.1016/j.neuroimage.2009.12.028, **PMID:** 20006715, **PMCID:** PMC2891595 20006715 10.1016/j.neuroimage.2009.12.028 PMC2891595

[bib53] Yu, Y., Herman, P., Rothman, D. L., Agarwal, D., & Hyder, F. (2018). Evaluating the gray and white matter energy budgets of human brain function. Journal of Cerebral Blood Flow and Metabolism, 38(8), 1339–1353. **DOI:** https://doi.org/10.1177/0271678X17708691, **PMID:** 28589753, **PMCID:** PMC6092772 28589753 10.1177/0271678X17708691 PMC6092772

[bib54] Zhu, X.-H., Qiao, H., Du, F., Xiong, Q., Liu, X., Zhang, X., … Chen, W. (2012). Quantitative imaging of energy expenditure in human brain. NeuroImage, 60(4), 2107–2117. **DOI:** https://doi.org/10.1016/j.neuroimage.2012.02.013, **PMID:** 22487547, **PMCID:** PMC3325488 22487547 10.1016/j.neuroimage.2012.02.013 PMC3325488

[bib55] Zoeller, D., Sandini, C., Schaer, M., Eliez, S., Bassett, D., & Van De Ville, D. (2019). Structural control energy of resting-state functional brain states reveals inefficient brain dynamics in psychosis vulnerability. bioRxiv:703561. 10.1101/703561.1101/703561 PMC8046160 33566395

