# Supplementary material for: The missing role of gray matter in studying brain controllability
Source: Netw Neurosci. 2021 Mar 1;5(1):198–210. doi: 10.1162/netn_a_00174 (PMC7935040; doi:10.1162/netn_a_00174)
Supplement: Supplementary file 1 [file netn-05-198-s001.pdf]

Jamalabadi, H., Zuberer, A., Kumar, V. J., Li, M., Alizadeh, S., Moradi, A. A., Gaser, C., Esterman, M., & Walter, M. (2021). Supporting information for "The missing role of gray matter in studying brain controllability." *Network Neuroscience*, *5*(1), 198–210. https://doi.org/10.1162/netn_a_00174

Supplementary Material: Modal controllability

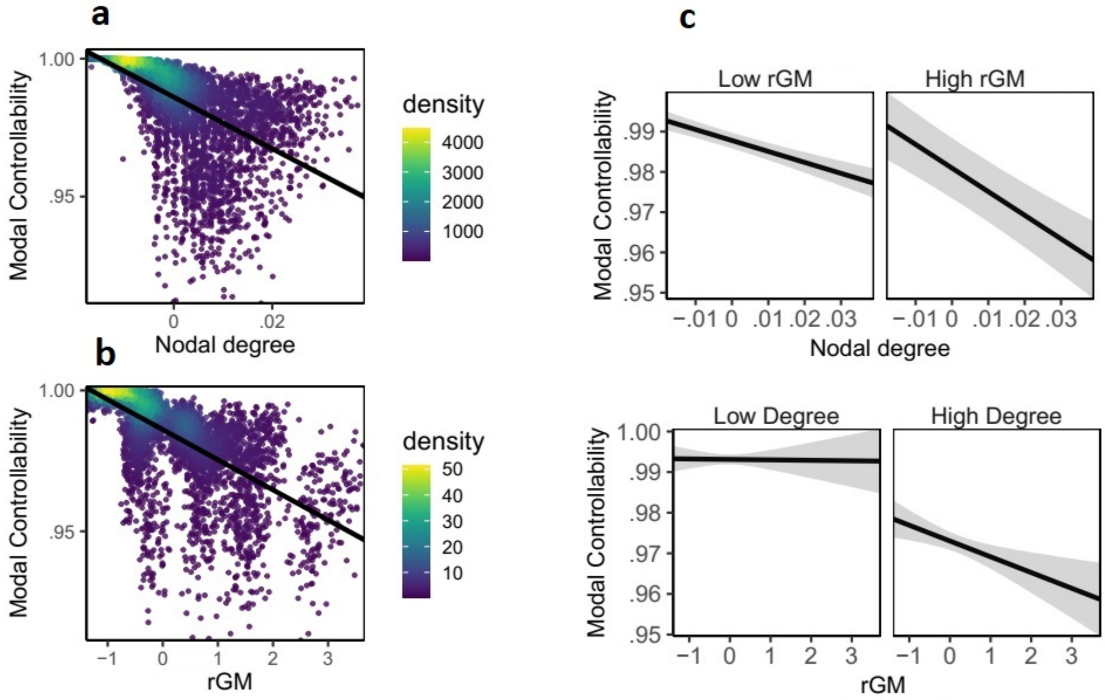

Figure B1: Visualization of interaction effect of nodal degree and rGM in the mixed effects model predicting modal controllability (MC) This effect was controlled for by the TIV and regional differences of modal controllability. Figure shows that MC is best explained by white matter structure and rGM together. Each dot represents one region from one subject. The density bar shows where the majority of the data is located. (A) Association between nodal degree and MC. (B) Association between rGM and MC. (C) Interaction between rGM and degree on MC suggests that highest levels of MC are reached when both degree and GM volume are high together. For visualization, median split was used to classify rGM and degree into high and low respectively. In the original model, both effects were preserved as continuous variables.

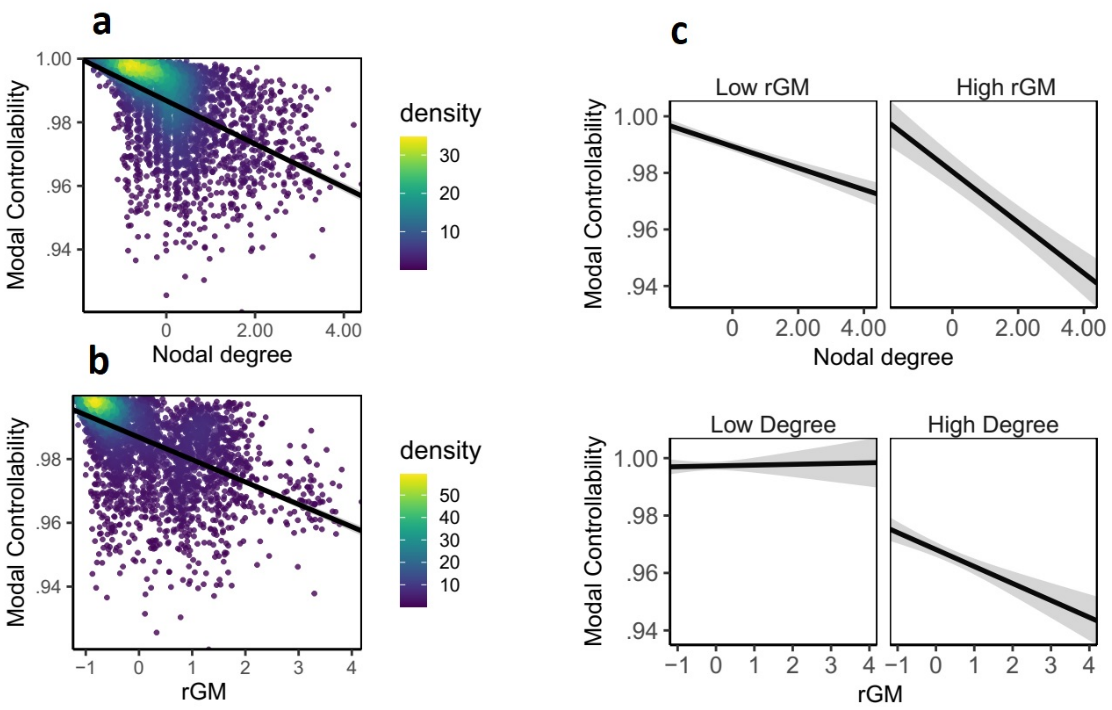

Figure B2: Replication sample. Visualization of interaction effect of nodal degree and rGM in the mixed effects model predicting modal controllability (MC) This effect was controlled for by TIV and regional differences of modal controllability. Figure shows that MC is best explained by white matter structure and rGM together. Each dot represents one region from one subject. The density bar shows where the majority of the data is located. (A) Association between nodal degree and MC. (B) Association between rGM and MC. (C) Interaction between rGM and regree on MC suggests that highest levels of MC are reached when both degree and rGM are high together. For visualization, median split was used to classify rGM and degree into high and low respectively. In the original model, both effects were preserved as continuous variables.
