# Supplementary material for: The missing role of gray matter in studying brain controllability
Source: Netw Neurosci. 2021 Mar 1;5(1):198–210. doi: 10.1162/netn_a_00174 (PMC7935040; doi:10.1162/netn_a_00174)
Supplement: Supplementary file 2 [file netn-05-198-s002.pdf]

Jamalabadi, H., Zuberer, A., Kumar, V. J., Li, M., Alizadeh, S., Moradi, A. A., Gaser, C., Esterman, M., & Walter, M. (2021). Supporting information for "The missing role of gray matter in studying brain controllability." *Network Neuroscience*, *5*(1), 198–210. https://doi.org/10.1162/netn_a_00174

Supplementary Material: Model comparisons

Table C1: Model comparison to select the best set of variables to explain variance of average controllability in Figure 1. In the formulation "TIV" represents whole brain volume, "rGM" refers to regional gray matter volume, and "regions" are the ROIs defined by the respective parcellation. "*" represents interaction term.

Test number	*Tested Models for Model I*	*AIC*	*LogLik*	*P(>Chisq)*
Test 1	AC ~ 1+(1|subjects)	15393	-7693	0.010
	AC ~ TIV+(1|subjects)	15389	-7690	
Test 2	AC ~ TIV+(1|subjects)	15389	-7690	2.2e-16
	AC ~ TIV + Regions + (1|subjects)	4970	-2388	
Test 3	AC ~ TIV + Regions + (1|subjects)	4970	-2388	2.2e-16
	AC ~ TIV + Regions + Nodal degree + (1|subjects)	4789	-2296	
Test 4	AC ~ TIV + Regions + Nodal degree + (1|subjects)	4789	-2296	0.119
	AC ~ TIV + Regions + Nodal degree + rGM+ (1|subjects)	4788	-2295	
Test 5	AC ~ TIV + Regions + Nodal degree + (1|subjects)	4789	-2296	0.010
	AC ~ TIV + Regions + Nodal degree * rGM + (1|subjects)	4783	-2291	
Test 6	AC ~ TIV + Regions + Nodal degree * rGM + (1|subjects)	4783	-2291	0.556
	AC ~ TIV + Regions + Nodal degree * rGM + Age + (1|subjects)	4785	-2291	
Test 7	AC ~ TIV + Regions + Nodal degree * rGM + (1|subjects)	4788	-2291	0.007
	AC ~ TIV + Regions + Nodal degree * rGM + sex + (1|subjects)	4778	-2288	

Table C2: Model comparison to select the best set of variables to predict average controllability in Figure 2. "TIV": total intracranial volume, "rGM": regional gray matter volume, and "Regions": ROIs in the parcellation of the data. "*" represents interaction term.

	Tested Models for Model II	*AIC*	*LogLik*	*P(>Chisq)*
Test 1	AC ~ TIV + Regions + Nodal degree +(1|subjects)	4989	-2296	2.2e-16
	AC ~ TIV + Regions* Nodal degree + (1|subjects)	4659	-2139	
Test 2	AC ~ TIV + Regions* Nodal degree + (1|subjects)	4659	-2139	0.1827
	AC ~ TIV + Regions* Nodal degree + rGM + (1|subjects)	4660	-2138	
Test 3	AC ~ TIV + Regions* Nodal degree + rGM + (1|subjects)	4660	-2138	1.459e-12
	AC ~ TIV + Regions * Nodal degree + Regions * rGM + (1|subjects)	4623	-2027	
Test 4	AC ~ TIV + Regions * Nodal degree + Regions * rGM + (1|subjects)	4623	-2027	0.595

<table>
<tr><td></td><td>AC ~ TIV + Regions * Nodal degree + Regions * rGM + age + (1|subjects)</td><td>4625</td><td>-2027</td><td></td></tr>
<tr><td rowspan="2">Test 5</td><td>AC ~ TIV + Regions * Nodal degree + Regions * rGM + (1|subjects)</td><td>4625</td><td>-2027</td><td rowspan="2">0.007</td></tr>
<tr><td>AC ~ TIV + Regions * Nodal degree + Regions * rGM + sex + (1|subjects)</td><td>4618</td><td>-2024</td></tr>
</table>
