# Supplementary material for: The missing role of gray matter in studying brain controllability
Source: Netw Neurosci. 2021 Mar 1;5(1):198–210. doi: 10.1162/netn_a_00174 (PMC7935040; doi:10.1162/netn_a_00174)
Supplement: Supplementary file 3 [file netn-05-198-s003.pdf]

Jamalabadi, H., Zuberer, A., Kumar, V. J., Li, M., Alizadeh, S., Moradi, A. A., Gaser, C., Esterman, M., & Walter, M. (2021). Supporting information for "The missing role of gray matter in studying brain controllability." *Network Neuroscience*, *5*(1), 198–210. https://doi.org/10.1162/netn_a_00174

Supplementary Material: Replication study methods

1.1 Data source and acquisition protocol

An open-access dataset, known as 'The Stockholm Sleepy Brain Study: Effects of Sleep Deprivation on Cognitive and Emotional Processing in Young and Old' (see more details in (Nilsonne et al., 2016) osf.io/bxfsb and https://openneuro.org/datasets/ds000201), was analyzed in the present study. In the original Stockholm Sleepy Brain Study, the effects of sleep deprivation on brain function with regard to emotional processing was investigated using a randomized cross-over design. The obtained anatomical, diffusion, and functional (at rest) magnetic resonance images were analyzed in the current study. After excluding the subjects with suspicious bval/bvecs tables extracted from the source DICOM files for diffusion imaging (also mentioned by the owners), 48 subjects with completed diffusion, anatomical and functional images were included in our study.

All subjects underwent diffusion MRI in a GE 3T (Discovery MR 750) scanner, using an 8-channel array head coil for signal reception and the built-in body coil for radiofrequency transmission.

Image acquisition was performed with a 3T MRI scanner (Discovery MR750, GE Medical Systems). The T1-weighted imaging with the following parameters (flip angle = 11°; TR = 64 ms; echo time TE = 2.8 ms for each image; 180 sagittal sections; spatial resolution 0.4688 x 0.46881 × 1 mm). The diffusion-weighted MRI (dMRI) acquisition protocol involved 5 non-diffusion weighted images (b-value 0 s/mm^2) and 45 non-collinear gradient directions (b-value 1000 s/mm^2) uniformly sampled over a sphere; *TE* = 80 ms, *TR* = 7000 ms, field of view FOV = 220 × 220 mm^2, imaging matrix = 96 × 96, 63 consecutive slices with a thickness of 2.3 mm.

With all image files in BIDS format (https://bids.neuroimaging.io/), the quality control was performed with the help of MRIQC output prior to all processing. MRIQC is an automated processing pipeline designed to compute and compare the image quality metrics for T1 weighted anatomical and T2* weighted functional scans in both single individual level and group level (see more details in https://mriqc.readthedocs.io).

1.2 Anatomical data preprocessing

First, in each subject, the T1-weighted (T1w) images were corrected for intensity non-uniformity (INU) with N4BiasFieldCorrection (Tustison et al., 2010) and then skull-stripped with the antsBrainExtraction.sh workflow ANTs 2.2.0 (Avants, Epstein, Grossman, & Gee, 2008). Next, spatial normalization to the ICBM 152 Nonlinear Asymmetrical template version 2009c (Fonov, Evans, McKinstry, Almli, & Collins, 2009) was performed through nonlinear registration with antsRegistration (ANTs 2.2.0), using brain-extracted versions of both T1w volume and template. Then, brain tissue segmentation of cerebrospinal fluid (CSF), white-matter (WM) and gray-matter (GM) was performed on the brain-extracted T1w using fast (FSL 5.0.9, RRID:SCR_002823, (Zhang, Brady, & Smith, 2001)). Afterwards, brain surfaces were reconstructed using recon-all (FreeSurfer 6.0.1, RRID:SCR_001847, (Dale, Fischl, & Sereno, 1999). The regional volumes, defined by the parcellation scheme (Aparc+Aseg, 84 rois), were extracted from the derived output of recon-all as well as the estimated intracranial volume. Additionally, the sub-cortical grey matter structures estimated by recon-all was replaced by the estimates from FSL's FIRST tool.

1.3 Diffusion data preprocessing

The diffusion data were preprocessed by MRtrix3 (http://www.mrtrix.org/) with an adapted pipeline from the structural connectome for Human Connectome Project (https://mrtrix.readthedocs.io/en/latest/quantitative_structural_connectivity/ismrm_hcp_tutorial.html) and Basic and Advanced Tractography with MRtrix for All Neurophiles (BATMAN, https://osf.io/fkyht/).

The mean B=0 image was calculated and used as the reference image for eddy current correction. The B1 field inhomogeneity correction for the DWI volume series and DWI data denoising by exploiting data redundancy in the PCA domain (Veraart2016a and Veraart2016b) were performed using Mrtrix3 commands: dwibiascorrect and dwidenosie, respectively. Within the estimated brain mask based on the mean b0 image, the response functions (RF) for the three different tissue types: white matter, gray matter, and CSF were estimated (Dhollander & Connelly, 2016). The tissue-specific RF was further

used as a kernel to perform constrained spherical deconvolution (CSD; (Tournier, Calamante, & Connelly, 2007; Tournier, Calamante, Gadian, & Connelly, 2004). Based on the derived RF for the different tissue-types, the Fiber Orientation Distribution (FOD, as explained in (Jeurissen, Tournier, Dhollander, Connelly, & Sijbers, 2014) crossing each voxel were generated (Tournier et al., 2007; Tournier et al., 2004)in each voxel. The voxels used for RF estimation of the different tissue types were displayed on the preprocessed DW-image and visually inspected in each subject as well as the derived FOD. Next, in order to create a whole-brain tractogram with biological plausibility of downstream streamline, the T1 image was registered to mean b0 image and further segmented into five tissue types for improving fiber tracking. After that, the probabilistic tractography using CSD algorithm in conjunction with Anatomically Constrained Tractography (ACT; Smith, Tournier, Calamante, & Connelly, 2012) based on the segmented five tissue types T1 was performed to create 1M streamlines in each subject. Given the bias towards streamline density of that straight track in regions of crossing fibers and the density of long tracks compared to short tracks is overestimated by CSD algorithm , the Spherical-deconvolution Informed Filtering of Tractograms (SIFT) algorithm (Smith, Tournier, Calamante, & Connelly, 2013) was applied to filters the tractograms and reduced the overall streamline count to 200k. In the end, we mapped the streamlines with the above derived Aparc+Aseg as the parcellated image to produce a connectome – “structural connectivity” matrix. The edge of this matrix was defined as the count how many tracks connect each region to the other regions within the Aparc+Aseg scheme.

1.4 References

Avants, B. B., Epstein, C. L., Grossman, M., & Gee, J. C. (2008). Symmetric diffeomorphic image registration with cross-correlation: evaluating automated labeling of elderly and neurodegenerative brain. *Medical image analysis, 12*(1), 26-41.

Dale, A. M., Fischl, B., & Sereno, M. I. (1999). Cortical surface-based analysis: I. Segmentation and surface reconstruction. *Neuroimage, 9*(2), 179-194.

Dhollander, T., & Connelly, A. (2016). *Generating a T1-like contrast using 3-tissue constrained spherical deconvolution results from single-shell (or multi-shell) diffusion MR data.* Paper presented at the ISMRM Workshop on Breaking the Barriers of Diffusion MRI. Lisbon, Portugal.

Fonov, V. S., Evans, A. C., McKinstry, R. C., Almli, C., & Collins, D. (2009). Unbiased nonlinear average age-appropriate brain templates from birth to adulthood. *Neuroimage*(47), S102.

Jeurissen, B., Tournier, J.-D., Dhollander, T., Connelly, A., & Sijbers, J. (2014). Multi-tissue constrained spherical deconvolution for improved analysis of multi-shell diffusion MRI data. *Neuroimage, 103*, 411-426.

Nilsonne, G., Tamm, S., d'Onofrio, P., Thuné, H. Å., Schwarz, J., Lavebratt, C., . . . Axelsson, J. (2016). A multimodal brain imaging dataset on sleep deprivation in young and old humans.

Smith, R. E., Tournier, J.-D., Calamante, F., & Connelly, A. (2012). Anatomically-constrained tractography: improved diffusion MRI streamlines tractography through effective use of anatomical information. *Neuroimage, 62*(3), 1924-1938.

Smith, R. E., Tournier, J.-D., Calamante, F., & Connelly, A. (2013). SIFT: spherical-deconvolution informed filtering of tractograms. *Neuroimage, 67*, 298-312.

Tournier, J.-D., Calamante, F., & Connelly, A. (2007). Robust determination of the fibre orientation distribution in diffusion MRI: non-negativity constrained super-resolved spherical deconvolution. *Neuroimage, 35*(4), 1459-1472.

Tournier, J.-D., Calamante, F., Gadian, D. G., & Connelly, A. (2004). Direct estimation of the fiber orientation density function from diffusion-weighted MRI data using spherical deconvolution. *Neuroimage, 23*(3), 1176-1185.

Tustison, N. J., Avants, B. B., Cook, P. A., Zheng, Y., Egan, A., Yushkevich, P. A., & Gee, J. C. (2010). N4ITK: improved N3 bias correction. *IEEE transactions on medical imaging, 29*(6), 1310.

Zhang, Y., Brady, M., & Smith, S. (2001). Segmentation of brain MR images through a hidden Markov random field model and the expectation-maximization algorithm. *IEEE transactions on medical imaging, 20*(1), 45-57.
