# Supplementary material for: The missing role of gray matter in studying brain controllability
Source: Netw Neurosci. 2021 Mar 1;5(1):198–210. doi: 10.1162/netn_a_00174 (PMC7935040; doi:10.1162/netn_a_00174)
Supplement: Supplementary file 4 [file netn-05-198-s004.pdf]

Jamalabadi, H., Zuberer, A., Kumar, V. J., Li, M., Alizadeh, S., Moradi, A. A., Gaser, C., Esterman, M., & Walter, M. (2021). Supporting information for “The missing role of gray matter in studying brain controllability.” *Network Neuroscience, 5*(1), 198–210. https://doi.org/10.1162/netn_a_00174

Table S1: details of the model in Figure 1

	**Average controllability**			**Modal controllability**		
Predictors	*std. Beta*	*standardized CI*	*p*	*std. Beta*	*standardized CI*	*p*
TIV	-0.01	-0.07 – 0.04	0.620	0.01	-0.04 – 0.06	0.662
NodalDegree	0.22	0.19 – 0.25	**<0.001**	-0.21	-0.24 – -0.18	**<0.001**
GMvolume	0.09	-0.04 – 0.21	0.173	-0.08	-0.20 – 0.03	0.156
roi [Amygdala_L]	-0.48	-0.67 – -0.30	0.184	0.53	0.35 – 0.70	0.103
roi [Amygdala_R]	-0.53	-0.71 – -0.35	0.132	0.56	0.39 – 0.73	0.074
roi [Angular_L]	-0.06	-0.17 – 0.06	0.773	0.05	-0.06 – 0.16	0.805
roi [Angular_R]	0.03	-0.08 – 0.14	0.528	-0.06	-0.16 – 0.05	0.495
roi [Calcarine_L]	2.11	1.83 – 2.39	**<0.001**	-2.14	-2.41 – -1.87	**<0.001**
roi [Calcarine_R]	2.64	2.34 – 2.95	**<0.001**	-2.60	-2.89 – -2.31	**<0.001**
roi [Caudate_L]	0.34	0.20 – 0.47	0.069	-0.32	-0.44 – -0.19	0.081
roi [Caudate_R]	-0.09	-0.21 – 0.04	0.865	0.06	-0.06 – 0.18	0.834
roi [Cingulate_Ant_L]	-0.35	-0.46 – -0.24	0.381	0.34	0.24 – 0.45	0.334
roi [Cingulate_Ant_R]	-0.41	-0.52 – -0.30	0.267	0.41	0.31 – 0.52	0.215
roi [Cingulate_Mid_L]	-0.12	-0.24 – -0.00	0.973	0.09	-0.03 – 0.20	0.929
roi [Cingulate_Mid_R]	0.22	0.09 – 0.35	0.170	-0.29	-0.41 – -0.16	0.105
roi [Cingulate_Post_L]	-0.37	-0.54 – -0.19	0.370	0.39	0.22 – 0.55	0.271
roi [Cingulate_Post_R]	-0.39	-0.58 – -0.21	0.320	0.42	0.24 – 0.60	0.220
roi [Cuneus_L]	2.20	2.06 – 2.34	**<0.001**	-2.13	-2.27 – -2.00	**<0.001**
roi [Cuneus_R]	2.39	2.15 – 2.63	**<0.001**	-2.34	-2.57 – -2.12	**<0.001**
roi [Frontal_Inf_Oper_L]	-0.25	-0.38 – -0.13	0.637	0.27	0.16 – 0.39	0.500
roi [Frontal_Inf_Oper_R]	-0.31	-0.42 – -0.20	0.477	0.32	0.22 – 0.43	0.383
roi [Frontal_Inf_Orb_2_L]	-0.46	-0.60 – -0.33	0.194	0.49	0.36 – 0.62	0.123
roi [Frontal_Inf_Orb_2_R]	-0.55	-0.69 – -0.41	0.103	0.58	0.44 – 0.71	0.058
roi [Frontal_Inf_Tri_L]	0.61	0.47 – 0.75	**0.004**	-0.55	-0.69 – -0.42	**0.007**
roi [Frontal_Inf_Tri_R]	-0.10	-0.21 – 0.01	0.903	0.10	-0.01 – 0.20	0.956
roi [Frontal_Med_Orb_L]	-0.53	-0.67 – -0.38	0.126	0.56	0.42 – 0.69	0.070

roi [Frontal_Med_Orb_R]	-0.57	-0.70 – -0.44	0.087	0.59	0.47 – 0.72	**0.049**
roi [Frontal_Mid_2_L]	2.48	1.97 – 3.00	**<0.001**	-2.54	-3.03 – -2.05	**<0.001**
roi [Frontal_Mid_2_R]	1.94	1.57 – 2.30	**<0.001**	-2.00	-2.35 – -1.65	**<0.001**
roi [Frontal_Sup_2_L]	1.76	1.16 – 2.35	**<0.001**	-1.82	-2.38 – -1.25	**<0.001**
roi [Frontal_Sup_2_R]	1.98	1.59 – 2.37	**<0.001**	-2.07	-2.44 – -1.70	**<0.001**
roi [Frontal_Sup_Medial_L]	0.25	0.08 – 0.42	0.142	-0.23	-0.40 – -0.07	0.168
roi [Frontal_Sup_Medial_R]	-0.12	-0.25 – 0.01	0.968	0.11	-0.01 – 0.23	0.988
roi [Fusiform_L]	0.06	-0.11 – 0.23	0.455	-0.08	-0.24 – 0.09	0.457
roi [Fusiform_R]	0.09	-0.10 – 0.27	0.410	-0.10	-0.28 – 0.07	0.400
roi [Heschl_L]	-0.43	-0.62 – -0.24	0.253	0.47	0.29 – 0.65	0.159
roi [Heschl_R]	-0.42	-0.61 – -0.23	0.281	0.45	0.27 – 0.63	0.181
roi [Hippocampus_L]	-0.40	-0.52 – -0.28	0.297	0.39	0.28 – 0.51	0.249
roi [Hippocampus_R]	-0.42	-0.54 – -0.29	0.262	0.41	0.29 – 0.53	0.217
roi [Insula_L]	-0.37	-0.50 – -0.25	0.336	0.36	0.24 – 0.48	0.306
roi [Insula_R]	-0.41	-0.53 – -0.30	0.262	0.40	0.28 – 0.51	0.240
roi [Lingual_L]	1.79	1.65 – 1.94	**<0.001**	-1.75	-1.89 – -1.61	**<0.001**
roi [Lingual_R]	2.04	1.88 – 2.21	**<0.001**	-1.98	-2.14 – -1.83	**<0.001**
roi [Occipital_Inf_L]	0.84	0.71 – 0.96	**<0.001**	-0.83	-0.95 – -0.71	**<0.001**
roi [Occipital_Inf_R]	0.17	0.05 – 0.29	0.242	-0.14	-0.26 – -0.02	0.305
roi [Occipital_Mid_L]	1.97	1.73 – 2.21	**<0.001**	-1.99	-2.22 – -1.76	**<0.001**
roi [Occipital_Mid_R]	1.90	1.75 – 2.04	**<0.001**	-1.85	-1.99 – -1.72	**<0.001**
roi [Occipital_Sup_L]	2.25	2.11 – 2.38	**<0.001**	-2.18	-2.31 – -2.05	**<0.001**
roi [Occipital_Sup_R]	2.35	2.18 – 2.51	**<0.001**	-2.26	-2.42 – -2.11	**<0.001**
roi [OFCant_L]	-0.45	-0.63 – -0.28	0.219	0.50	0.33 – 0.67	0.122
roi [OFCant_R]	-0.46	-0.63 – -0.30	0.205	0.51	0.35 – 0.66	0.114
roi [OFClat_L]	-0.43	-0.63 – -0.23	0.267	0.48	0.29 – 0.66	0.155
roi [OFClat_R]	-0.41	-0.62 – -0.20	0.299	0.46	0.26 – 0.66	0.176
roi [OFCmed_L]	-0.45	-0.62 – -0.28	0.224	0.49	0.34 – 0.65	0.126

roi [OFCmed_R]	-0.45	-0.61 – -0.29	0.223	0.49	0.34 – 0.65	0.125
roi [OFCpost_L]	-0.49	-0.65 – -0.34	0.164	0.53	0.38 – 0.68	0.091
roi [OFCpost_R]	-0.48	-0.64 – -0.32	0.180	0.52	0.37 – 0.68	0.100
roi [Olfactory_L]	-0.49	-0.67 – -0.30	0.180	0.53	0.35 – 0.70	0.101
roi [Olfactory_R]	-0.50	-0.68 – -0.33	0.156	0.54	0.37 – 0.71	0.088
roi [Pallidum_L]	-0.35	-0.55 – -0.16	0.402	0.39	0.20 – 0.58	0.284
roi [Pallidum_R]	-0.42	-0.61 – -0.23	0.275	0.45	0.27 – 0.63	0.184
roi [Paracentral_Lobule_L]	-0.28	-0.40 – -0.16	0.563	0.29	0.17 – 0.40	0.469
roi [Paracentral_Lobule_R]	-0.35	-0.49 – -0.20	0.401	0.36	0.23 – 0.50	0.304
roi [ParaHippocampal_L]	-0.37	-0.49 – -0.25	0.347	0.38	0.27 – 0.50	0.262
roi [ParaHippocampal_R]	-0.30	-0.41 – -0.19	0.498	0.30	0.20 – 0.41	0.427
roi [Parietal_Inf_L]	0.65	0.50 – 0.80	**0.002**	-0.71	-0.85 – -0.56	**0.001**
roi [Parietal_Inf_R]	0.12	0.01 – 0.23	0.328	-0.16	-0.26 – -0.05	0.271
roi [Parietal_Sup_L]	0.27	0.15 – 0.38	0.115	-0.29	-0.40 – -0.18	0.097
roi [Parietal_Sup_R]	-0.17	-0.28 – -0.06	0.878	0.16	0.05 – 0.27	0.831
roi [Postcentral_L]	2.03	1.78 – 2.29	**<0.001**	-2.10	-2.34 – -1.86	**<0.001**
roi [Postcentral_R]	1.54	1.31 – 1.77	**<0.001**	-1.64	-1.86 – -1.43	**<0.001**
roi [Precentral_L]	1.99	1.76 – 2.22	**<0.001**	-1.96	-2.18 – -1.74	**<0.001**
roi [Precentral_R]	1.80	1.60 – 1.99	**<0.001**	-1.83	-2.02 – -1.65	**<0.001**
roi [Precuneus_L]	0.20	-0.05 – 0.45	0.230	-0.22	-0.46 – 0.01	0.205
roi [Precuneus_R]	0.51	0.29 – 0.73	**0.018**	-0.46	-0.68 – -0.25	**0.026**
roi [Putamen_L]	0.20	0.05 – 0.35	0.202	-0.17	-0.32 – -0.03	0.251
roi [Putamen_R]	-0.41	-0.56 – -0.27	0.271	0.39	0.25 – 0.53	0.257
roi [Rectus_L]	-0.52	-0.65 – -0.38	0.133	0.55	0.42 – 0.68	0.072
roi [Rectus_R]	-0.50	-0.64 – -0.36	0.148	0.54	0.40 – 0.67	0.082
roi [Rolandic_Oper_L]	-0.36	-0.48 – -0.24	0.374	0.36	0.24 – 0.48	0.305
roi [Rolandic_Oper_R]	-0.43	-0.54 – -0.32	0.233	0.44	0.33 – 0.54	0.176
roi [Supp_Motor_Area_L]	-0.22	-0.35 – -0.09	0.720	0.25	0.13 – 0.37	0.563

roi [Supp_Motor_Area_R]	0.12	-0.02 – 0.26	0.328	-0.14	-0.27 – -0.01	0.309
roi [SupraMarginal_L]	-0.21	-0.32 – -0.10	0.751	0.21	0.10 – 0.32	0.683
roi [SupraMarginal_R]	-0.03	-0.15 – 0.08	0.707	-0.00	-0.11 – 0.11	0.650
roi [Temporal_Inf_L]	-0.03	-0.28 – 0.21	0.721	-0.00	-0.24 – 0.23	0.666
roi [Temporal_Inf_R]	0.23	-0.04 – 0.50	0.196	-0.28	-0.53 – -0.02	0.148
roi [Temporal_Mid_L]	0.82	0.40 – 1.23	**0.003**	-0.93	-1.33 – -0.53	**0.001**
roi [Temporal_Mid_R]	1.08	0.73 – 1.44	**<0.001**	-1.19	-1.53 – -0.86	**<0.001**
roi [Temporal_Pole_Mid_L]	-0.50	-0.64 – -0.36	0.147	0.54	0.41 – 0.68	0.078
roi [Temporal_Pole_Mid_R]	-0.55	-0.67 – -0.42	0.100	0.59	0.47 – 0.70	0.052
roi [Temporal_Pole_Sup_L]	-0.56	-0.67 – -0.44	0.091	0.59	0.48 – 0.70	**0.047**
roi [Temporal_Pole_Sup_R]	-0.56	-0.67 – -0.44	0.093	0.59	0.47 – 0.70	0.050
roi [Temporal_Sup_L]	0.24	0.09 – 0.38	0.154	-0.30	-0.44 – -0.16	0.093
roi [Temporal_Sup_R]	0.08	-0.12 – 0.28	0.429	-0.13	-0.32 – 0.06	0.340
roi [Thalamus_L]	0.37	0.21 – 0.53	0.055	-0.34	-0.49 – -0.19	0.070
roi [Thalamus_R]	0.10	-0.05 – 0.26	0.374	-0.12	-0.26 – 0.03	0.363
sex [male]	-0.15	-0.26 – -0.04	**0.007**	0.14	0.03 – 0.24	**0.009**
NodalDegree * GMvolume	0.04	0.01 – 0.07	**0.010**	-0.04	-0.07 – -0.01	**0.006**
**Random Effects**						
σ^2	0.13			0.12		
τ_{00}	0.03 subjects			0.02 subjects		
N	65 subjects			65 subjects		
Observations	5462			5462		

Note. Results of linear mixed effect model predicting average controllability based on the interaction between nodal degree and regional gray matter volume. Region index extracted from AAL2 atlas and the TIV were additional predictors. σ_2: random effect variance; r_{00} : between-subject variance; CI: bootstrapped 95% confidence intervals. p-values were computed via Wald-statistics approximation (treating t as Wald z) and Bonferroni corrected.

Table S2: details of the model in Figure 2

	Average controllability			Modal controllability		
Predictors	*std. Beta*	*standardized CI*	*p*	*std. Beta*	*standardized CI*	*p*
(Intercept)	0.23	0.15 – 0.32	0.122	-0.23	-0.31 – -0.15	0.127
TIV	-0.02	-0.08 – 0.04	0.515	0.02	-0.04 – 0.07	0.547
sex [male]	-0.14	-0.26 – -0.03	**0.014**	0.13	0.02 – 0.25	**0.018**
NodalDegree : roi [Amygdala_L]	-0.07	-0.50 – 0.36	0.758	0.06	-0.35 – 0.48	0.769
NodalDegree : roi [Amygdala_R]	0.16	-0.36 – 0.68	0.539	-0.15	-0.65 – 0.34	0.546
NodalDegree : roi [Angular_L]	0.58	0.16 – 1.01	**0.007**	-0.59	-0.99 – -0.19	**0.004**
NodalDegree : roi [Angular_R]	0.40	0.02 – 0.77	**0.037**	-0.40	-0.76 – -0.04	**0.027**
NodalDegree : roi [Calcarine_L]	1.78	0.69 – 2.86	**0.001**	-1.82	-2.86 – -0.79	**0.001**
NodalDegree : roi [Calcarine_R]	0.36	-0.37 – 1.09	0.330	-0.44	-1.14 – 0.25	0.211
NodalDegree : roi [Caudate_L]	0.54	0.40 – 0.67	**<0.001**	-0.50	-0.63 – -0.38	**<0.001**
NodalDegree : roi [Caudate_R]	0.10	-0.12 – 0.32	0.371	-0.08	-0.29 – 0.13	0.464
NodalDegree : roi [Cingulate_Ant_L]	0.53	0.20 – 0.85	**0.001**	-0.52	-0.82 – -0.21	**0.001**
NodalDegree : roi [Cingulate_Ant_R]	0.50	0.09 – 0.91	**0.016**	-0.49	-0.88 – -0.10	**0.014**
NodalDegree : roi [Cingulate_Mid_L]	0.13	-0.21 – 0.47	0.440	-0.12	-0.45 – 0.20	0.457
NodalDegree : roi [Cingulate_Mid_R]	0.29	-0.03 – 0.60	0.072	-0.29	-0.59 – 0.01	0.057
NodalDegree : roi [Cingulate_Post_L]	0.09	-0.24 – 0.41	0.596	-0.09	-0.40 – 0.22	0.569
NodalDegree : roi [Cingulate_Post_R]	0.08	-0.25 – 0.41	0.625	-0.08	-0.39 – 0.23	0.615
NodalDegree : roi [Cuneus_L]	-2.70	-3.33 – -2.08	**<0.001**	2.60	2.00 – 3.20	**<0.001**

NodalDegree : roi [Cuneus_R]	-1.34	-2.11 – -0.57	**0.001**	1.27	0.53 – 2.00	**0.001**
NodalDegree : roi [Frontal_Inf_Oper_L]	0.16	-0.16 – 0.47	0.327	-0.14	-0.44 – 0.16	0.351
NodalDegree : roi [Frontal_Inf_Oper_R]	0.07	-0.23 – 0.36	0.657	-0.05	-0.34 – 0.23	0.704
NodalDegree : roi [Frontal_Inf_Orb_2_L]	0.28	-0.27 – 0.83	0.320	-0.28	-0.81 – 0.25	0.296
NodalDegree : roi [Frontal_Inf_Orb_2_R]	0.14	-0.36 – 0.64	0.587	-0.13	-0.61 – 0.34	0.582
NodalDegree : roi [Frontal_Inf_Tri_L]	0.26	-0.04 – 0.56	0.090	-0.26	-0.55 – 0.02	0.073
NodalDegree : roi [Frontal_Inf_Tri_R]	0.80	0.34 – 1.27	**0.001**	-0.80	-1.24 – -0.36	**<0.001**
NodalDegree : roi [Frontal_Med_Orb_L]	-0.11	-0.55 – 0.32	0.611	0.10	-0.31 – 0.52	0.631
NodalDegree : roi [Frontal_Med_Orb_R]	0.21	-0.15 – 0.58	0.253	-0.22	-0.57 – 0.14	0.228
NodalDegree : roi [Frontal_Mid_2_L]	15.11	4.09 – 26.13	**0.007**	-14.70	-25.22 – -4.17	**0.006**
NodalDegree : roi [Frontal_Mid_2_R]	0.41	0.15 – 0.66	**0.002**	-0.38	-0.62 – -0.13	**0.003**
NodalDegree : roi [Frontal_Sup_2_L]	3.01	0.24 – 5.78	**0.033**	-2.87	-5.52 – -0.22	**0.034**
NodalDegree : roi [Frontal_Sup_2_R]	0.21	-0.25 – 0.67	0.363	-0.22	-0.66 – 0.22	0.334
NodalDegree : roi [Frontal_Sup_Medial_L]	-0.26	-0.69 – 0.18	0.248	0.24	-0.17 – 0.66	0.253
NodalDegree : roi [Frontal_Sup_Medial_R]	0.22	-0.15 – 0.59	0.243	-0.22	-0.57 – 0.14	0.230
NodalDegree : roi [Fusiform_L]	0.41	-0.18 – 0.99	0.171	-0.37	-0.93 – 0.18	0.187
NodalDegree : roi [Fusiform_R]	0.62	0.19 – 1.04	**0.005**	-0.59	-1.00 – -0.18	**0.005**
NodalDegree : roi [Heschl_L]	-0.09	-0.87 – 0.69	0.825	0.07	-0.68 – 0.81	0.862
	0.25	-0.28 – 0.78	0.357	-0.25	-0.76 – 0.25	0.327

NodalDegree : roi [Heschl_R]						
NodalDegree : roi [Hippocampus_L]	-0.06	-0.39 – 0.26	0.693	0.06	-0.24 – 0.37	0.681
NodalDegree : roi [Hippocampus_R]	0.02	-0.31 – 0.35	0.915	-0.00	-0.32 – 0.31	0.975
NodalDegree : roi [Insula_L]	-0.07	-0.28 – 0.15	0.535	0.08	-0.12 – 0.29	0.419
NodalDegree : roi [Insula_R]	0.07	-0.27 – 0.41	0.683	-0.08	-0.41 – 0.25	0.635
NodalDegree : roi [Lingual_L]	0.78	0.38 – 1.17	**<0.001**	-0.69	-1.07 – -0.31	**<0.001**
NodalDegree : roi [Lingual_R]	-0.76	-1.20 – -0.32	**0.001**	0.75	0.33 – 1.17	**<0.001**
NodalDegree : roi [Occipital_Inf_L]	1.24	0.60 – 1.89	**<0.001**	-1.13	-1.75 – -0.51	**<0.001**
NodalDegree : roi [Occipital_Inf_R]	0.83	0.17 – 1.48	**0.014**	-0.77	-1.40 – -0.14	**0.016**
NodalDegree : roi [Occipital_Mid_L]	-0.41	-0.94 – 0.11	0.125	0.38	-0.12 – 0.88	0.137
NodalDegree : roi [Occipital_Mid_R]	1.54	0.99 – 2.09	**<0.001**	-1.43	-1.96 – -0.91	**<0.001**
NodalDegree : roi [Occipital_Sup_L]	-0.18	-0.67 – 0.32	0.480	0.15	-0.32 – 0.62	0.538
NodalDegree : roi [Occipital_Sup_R]	0.68	0.21 – 1.16	**0.005**	-0.65	-1.11 – -0.20	**0.005**
NodalDegree : roi [OFCant_L]	-0.03	-0.40 – 0.33	0.864	0.04	-0.31 – 0.38	0.842
NodalDegree : roi [OFCant_R]	-0.08	-0.46 – 0.30	0.684	0.08	-0.29 – 0.44	0.678
NodalDegree : roi [OFClat_L]	0.29	-0.44 – 1.02	0.439	-0.28	-0.98 – 0.42	0.431
NodalDegree : roi [OFClat_R]	0.09	-0.99 – 1.17	0.867	-0.12	-1.15 – 0.92	0.826
NodalDegree : roi [OFCmed_L]	0.07	-0.29 – 0.42	0.701	-0.08	-0.42 – 0.26	0.661
NodalDegree : roi [OFCmed_R]	-0.05	-0.43 – 0.34	0.811	0.04	-0.33 – 0.40	0.845

NodalDegree : roi [OFCpost_L]	0.10	-0.28 – 0.47	0.607	-0.10	-0.46 – 0.26	0.592
NodalDegree : roi [OFCpost_R]	0.01	-0.48 – 0.51	0.965	-0.01	-0.48 – 0.46	0.961
NodalDegree : roi [Olfactory_L]	0.14	-0.46 – 0.75	0.644	-0.16	-0.74 – 0.42	0.593
NodalDegree : roi [Olfactory_R]	0.27	-0.33 – 0.88	0.376	-0.28	-0.85 – 0.30	0.352
NodalDegree : roi [Pallidum_L]	0.32	-0.12 – 0.75	0.157	-0.33	-0.75 – 0.09	0.120
NodalDegree : roi [Pallidum_R]	0.27	-0.12 – 0.66	0.170	-0.27	-0.64 – 0.09	0.144
NodalDegree : roi [Paracentral_Lobule_L]	0.43	0.06 – 0.79	**0.021**	-0.42	-0.77 – -0.08	**0.016**
NodalDegree : roi [Paracentral_Lobule_R]	0.41	-0.07 – 0.89	0.093	-0.43	-0.89 – 0.04	0.070
NodalDegree : roi [ParaHippocampal_L]	0.08	-0.42 – 0.59	0.752	-0.09	-0.58 – 0.39	0.707
NodalDegree : roi [ParaHippocampal_R]	0.53	0.16 – 0.90	**0.005**	-0.54	-0.89 – -0.18	**0.003**
NodalDegree : roi [Parietal_Inf_L]	0.39	0.07 – 0.72	**0.018**	-0.38	-0.70 – -0.07	**0.016**
NodalDegree : roi [Parietal_Inf_R]	0.24	-0.09 – 0.57	0.147	-0.20	-0.51 – 0.12	0.219
NodalDegree : roi [Parietal_Sup_L]	0.30	0.03 – 0.58	**0.031**	-0.30	-0.56 – -0.04	**0.024**
NodalDegree : roi [Parietal_Sup_R]	0.05	-0.25 – 0.36	0.731	-0.05	-0.34 – 0.24	0.738
NodalDegree : roi [Postcentral_L]	0.19	-0.27 – 0.64	0.422	-0.15	-0.58 – 0.28	0.502
NodalDegree : roi [Postcentral_R]	0.33	-0.14 – 0.81	0.170	-0.31	-0.76 – 0.15	0.187
NodalDegree : roi [Precentral_L]	0.05	-0.34 – 0.43	0.818	-0.06	-0.43 – 0.31	0.760
NodalDegree : roi [Precentral_R]	0.09	-0.17 – 0.35	0.495	-0.11	-0.36 – 0.14	0.388
	0.40	0.16 – 0.65	**0.001**	-0.38		**0.002**

NodalDegree : roi [Precuneus_L]					-0.61 – -0.14	
NodalDegree : roi [Precuneus_R]	0.18	-0.02 – 0.38	0.078	-0.17	-0.37 – 0.02	0.074
NodalDegree : roi [Putamen_L]	0.06	-0.08 – 0.19	0.411	-0.05	-0.18 – 0.08	0.449
NodalDegree : roi [Putamen_R]	-0.08	-0.22 – 0.07	0.290	0.08	-0.05 – 0.22	0.240
NodalDegree : roi [Rectus_L]	0.15	-0.20 – 0.50	0.412	-0.16	-0.49 – 0.18	0.362
NodalDegree : roi [Rectus_R]	0.18	-0.21 – 0.57	0.366	-0.20	-0.57 – 0.18	0.300
NodalDegree : roi [Rolandic_Oper_L]	0.18	-0.21 – 0.56	0.376	-0.18	-0.55 – 0.19	0.346
NodalDegree : roi [Rolandic_Oper_R]	0.25	-0.25 – 0.76	0.324	-0.27	-0.76 – 0.21	0.268
NodalDegree : roi [Supp_Motor_Area_L]	-0.05	-0.27 – 0.17	0.648	0.06	-0.15 – 0.27	0.571
NodalDegree : roi [Supp_Motor_Area_R]	0.09	-0.17 – 0.34	0.507	-0.09	-0.33 – 0.15	0.465
NodalDegree : roi [SupraMarginal_L]	0.18	-0.35 – 0.72	0.502	-0.21	-0.72 – 0.30	0.420
NodalDegree : roi [SupraMarginal_R]	0.09	-0.27 – 0.45	0.612	-0.12	-0.46 – 0.23	0.510
NodalDegree : roi [Temporal_Inf_L]	0.19	-0.10 – 0.49	0.192	-0.19	-0.47 – 0.09	0.174
NodalDegree : roi [Temporal_Inf_R]	0.67	0.37 – 0.98	**<0.001**	-0.67	-0.97 – -0.38	**<0.001**
NodalDegree : roi [Temporal_Mid_L]	0.63	0.42 – 0.84	**<0.001**	-0.62	-0.83 – -0.42	**<0.001**
NodalDegree : roi [Temporal_Mid_R]	0.58	0.32 – 0.85	**<0.001**	-0.58	-0.83 – -0.33	**<0.001**
NodalDegree : roi [Temporal_Pole_Mid_L]	0.13	-0.28 – 0.53	0.542	-0.12	-0.51 – 0.27	0.540
NodalDegree : roi [Temporal_Pole_Mid_R]	0.32	-0.06 – 0.69	0.096	-0.32	-0.68 – 0.03	0.074
NodalDegree : roi [Temporal_Pole_Sup_L]	0.80	0.43 – 1.16	**<0.001**	-0.81	-1.16 – -0.46	**<0.001**

NodalDegree : roi [Temporal_Pole_Sup_R]	0.27	-0.08 – 0.62	0.137	-0.28	-0.62 – 0.06	0.104
NodalDegree : roi [Temporal_Sup_L]	0.14	-0.18 – 0.47	0.384	-0.14	-0.45 – 0.17	0.369
NodalDegree : roi [Temporal_Sup_R]	0.37	0.02 – 0.72	**0.039**	-0.37	-0.70 – -0.03	**0.031**
NodalDegree : roi [Thalamus_L]	0.71	0.45 – 0.96	**<0.001**	-0.63	-0.88 – -0.39	**<0.001**
NodalDegree : roi [Thalamus_R]	0.45	0.19 – 0.71	**0.001**	-0.43	-0.67 – -0.18	**0.001**
roi [Amygdala_L] : GMvolume	0.94	0.52 – 1.37	**<0.001**	-0.96	-1.37 – -0.55	**<0.001**
roi [Amygdala_R] : GMvolume	0.77	0.45 – 1.09	**<0.001**	-0.80	-1.10 – -0.49	**<0.001**
roi [Angular_L] : GMvolume	0.42	-0.35 – 1.19	0.287	-0.36	-1.10 – 0.37	0.333
roi [Angular_R] : GMvolume	-0.78	-1.25 – -0.30	**0.001**	0.70	0.24 – 1.15	**0.003**
roi [Calcarine_L] : GMvolume	1.77	1.37 – 2.16	**<0.001**	-1.79	-2.17 – -1.41	**<0.001**
roi [Calcarine_R] : GMvolume	5.61	4.50 – 6.73	**<0.001**	-5.41	-6.47 – -4.34	**<0.001**
roi [Caudate_L] : GMvolume	0.79	0.39 – 1.19	**<0.001**	-0.76	-1.14 – -0.38	**<0.001**
roi [Caudate_R] : GMvolume	0.55	-0.01 – 1.11	0.053	-0.46	-0.99 – 0.07	0.090
roi [Cingulate_Ant_L] : GMvolume	3.76	2.61 – 4.91	**<0.001**	-3.73	-4.83 – -2.63	**<0.001**
roi [Cingulate_Ant_R] : GMvolume	2.88	2.28 – 3.48	**<0.001**	-2.87	-3.45 – -2.30	**<0.001**
roi [Cingulate_Mid_L] : GMvolume	-0.71	-0.96 – -0.45	**<0.001**	0.63	0.39 – 0.87	**<0.001**
roi [Cingulate_Mid_R] : GMvolume	0.05	-0.16 – 0.26	0.647	-0.14	-0.35 – 0.06	0.168
roi [Cingulate_Post_L] : GMvolume	0.72	0.48 – 0.96	**<0.001**	-0.73	-0.96 – -0.50	**<0.001**
	0.66	0.44 – 0.88	**<0.001**	-0.68		**<0.001**

roi [Cingulate_Post_R] : GMvolume					-0.89 – -0.47	
roi [Cuneus_L] : GMvolume	-0.84	-2.49 – 0.80	0.317	0.78	-0.80 – 2.35	0.334
roi [Cuneus_R] : GMvolume	-16.17	-18.46 – -13.88	**<0.001**	15.97	13.77 – 18.16	**<0.001**
roi [Frontal_Inf_Oper_L] : GMvolume	1.00	0.75 – 1.24	**<0.001**	-1.04	-1.27 – -0.81	**<0.001**
roi [Frontal_Inf_Oper_R] : GMvolume	2.24	1.76 – 2.73	**<0.001**	-2.28	-2.75 – -1.82	**<0.001**
roi [Frontal_Inf_Orb_2_L] : GMvolume	1.07	0.81 – 1.32	**<0.001**	-1.10	-1.34 – -0.85	**<0.001**
roi [Frontal_Inf_Orb_2_R] : GMvolume	1.23	0.92 – 1.55	**<0.001**	-1.27	-1.57 – -0.96	**<0.001**
roi [Frontal_Inf_Tri_L] : GMvolume	0.58	0.34 – 0.82	**<0.001**	-0.50	-0.73 – -0.27	**<0.001**
roi [Frontal_Inf_Tri_R] : GMvolume	-0.79	-1.32 – -0.25	**0.004**	0.76	0.25 – 1.28	**0.004**
roi [Frontal_Med_Orb_L] : GMvolume	1.42	0.97 – 1.86	**<0.001**	-1.44	-1.86 – -1.01	**<0.001**
roi [Frontal_Med_Orb_R] : GMvolume	1.37	1.09 – 1.65	**<0.001**	-1.40	-1.67 – -1.13	**<0.001**
roi [Frontal_Mid_2_L] : GMvolume	-3.34	-6.62 – -0.07	**0.045**	3.20	0.07 – 6.33	**0.045**
roi [Frontal_Mid_2_R] : GMvolume	0.67	0.54 – 0.81	**<0.001**	-0.71	-0.83 – -0.58	**<0.001**
roi [Frontal_Sup_2_L] : GMvolume	-0.59	-1.98 – 0.80	0.405	0.50	-0.83 – 1.83	0.459
roi [Frontal_Sup_2_R] : GMvolume	0.82	0.52 – 1.11	**<0.001**	-0.84	-1.12 – -0.56	**<0.001**
roi [Frontal_Sup_Medial_L] : GMvolume	0.63	0.18 – 1.07	**0.006**	-0.58	-1.01 – -0.16	**0.007**
roi [Frontal_Sup_Medial_R] : GMvolume	-0.59	-1.27 – 0.09	0.090	0.59	-0.06 – 1.24	0.076
roi [Fusiform_L] : GMvolume	-0.04	-0.20 – 0.12	0.628	0.04	-0.12 – 0.19	0.662

roi [Fusiform_R] : GMvolume	-0.06	-0.16 – 0.03	0.190	0.05	-0.04 – 0.14	0.275
roi [Heschl_L] : GMvolume	0.89	0.18 – 1.59	**0.013**	-0.89	-1.56 – -0.21	**0.010**
roi [Heschl_R] : GMvolume	0.58	0.06 – 1.10	**0.030**	-0.59	-1.09 – -0.09	**0.020**
roi [Hippocampus_L] : GMvolume	1.55	1.29 – 1.81	**<0.001**	-1.54	-1.78 – -1.29	**<0.001**
roi [Hippocampus_R] : GMvolume	1.43	1.20 – 1.66	**<0.001**	-1.42	-1.64 – -1.20	**<0.001**
roi [Insula_L] : GMvolume	-0.50	-0.92 – -0.08	**0.021**	0.46	0.05 – 0.86	**0.026**
roi [Insula_R] : GMvolume	-1.35	-1.69 – -1.02	**<0.001**	1.32	1.00 – 1.64	**<0.001**
roi [Lingual_L] : GMvolume	1.97	1.64 – 2.29	**<0.001**	-1.96	-2.27 – -1.65	**<0.001**
roi [Lingual_R] : GMvolume	2.98	2.63 – 3.33	**<0.001**	-2.90	-3.23 – -2.56	**<0.001**
roi [Occipital_Inf_L] : GMvolume	-1.84	-2.37 – -1.31	**<0.001**	1.75	1.24 – 2.26	**<0.001**
roi [Occipital_Inf_R] : GMvolume	-0.13	-0.61 – 0.35	0.588	0.05	-0.41 – 0.51	0.829
roi [Occipital_Mid_L] : GMvolume	1.68	1.30 – 2.06	**<0.001**	-1.67	-2.03 – -1.30	**<0.001**
roi [Occipital_Mid_R] : GMvolume	1.61	1.01 – 2.22	**<0.001**	-1.64	-2.22 – -1.07	**<0.001**
roi [Occipital_Sup_L] : GMvolume	-6.93	-7.46 – -6.40	**<0.001**	6.71	6.20 – 7.21	**<0.001**
roi [Occipital_Sup_R] : GMvolume	-8.78	-9.81 – -7.76	**<0.001**	8.42	7.44 – 9.39	**<0.001**
roi [OFCant_L] : GMvolume	1.07	0.56 – 1.58	**<0.001**	-1.11	-1.59 – -0.62	**<0.001**
roi [OFCant_R] : GMvolume	1.28	0.69 – 1.87	**<0.001**	-1.31	-1.87 – -0.75	**<0.001**
roi [OFClat_L] : GMvolume	0.50	-0.41 – 1.41	0.285	-0.53	-1.40 – 0.34	0.231
roi [OFClat_R] : GMvolume	0.70	-0.66 – 2.06	0.316	-0.69	-1.99 – 0.61	0.298
	1.04	0.47 – 1.60	**<0.001**	-1.06		**<0.001**

roi [OFCmed_L] : GMvolume					-1.60 – -0.52	
roi [OFCmed_R] : GMvolume	1.29	0.63 – 1.95	**<0.001**	-1.31	-1.94 – -0.68	**<0.001**
roi [OFCpost_L] : GMvolume	1.07	0.59 – 1.55	**<0.001**	-1.10	-1.56 – -0.64	**<0.001**
roi [OFCpost_R] : GMvolume	1.11	0.46 – 1.75	**0.001**	-1.13	-1.75 – -0.52	**<0.001**
roi [Olfactory_L] : GMvolume	0.77	0.17 – 1.37	**0.012**	-0.78	-1.35 – -0.21	**0.008**
roi [Olfactory_R] : GMvolume	0.69	0.18 – 1.20	**0.008**	-0.71	-1.20 – -0.22	**0.004**
roi [Pallidum_L] : GMvolume	0.43	0.11 – 0.76	**0.010**	-0.44	-0.75 – -0.12	**0.006**
roi [Pallidum_R] : GMvolume	0.54	0.28 – 0.81	**<0.001**	-0.56	-0.81 – -0.31	**<0.001**
roi [Paracentral_Lobule_L] : GMvolume	1.37	1.03 – 1.72	**<0.001**	-1.40	-1.72 – -1.07	**<0.001**
roi [Paracentral_Lobule_R] : GMvolume	0.73	0.46 – 1.01	**<0.001**	-0.74	-1.01 – -0.48	**<0.001**
roi [ParaHippocampal_L] : GMvolume	1.93	0.84 – 3.01	**<0.001**	-1.92	-2.95 – -0.88	**<0.001**
roi [ParaHippocampal_R] : GMvolume	2.12	0.56 – 3.67	**0.008**	-2.05	-3.54 – -0.57	**0.007**
roi [Parietal_Inf_L] : GMvolume	0.51	0.32 – 0.69	**<0.001**	-0.57	-0.75 – -0.40	**<0.001**
roi [Parietal_Inf_R] : GMvolume	1.16	-0.23 – 2.55	0.103	-1.00	-2.33 – 0.33	0.141
roi [Parietal_Sup_L] : GMvolume	0.18	-0.21 – 0.57	0.356	-0.25	-0.62 – 0.12	0.182
roi [Parietal_Sup_R] : GMvolume	-0.87	-1.28 – -0.46	**<0.001**	0.85	0.46 – 1.24	**<0.001**
roi [Postcentral_L] : GMvolume	1.14	0.95 – 1.32	**<0.001**	-1.19	-1.36 – -1.01	**<0.001**
roi [Postcentral_R] : GMvolume	0.88	0.67 – 1.10	**<0.001**	-0.95	-1.16 – -0.75	**<0.001**

roi [Precentral_L] : GMvolume	1.52	1.13 – 1.90	**<0.001**	-1.47	-1.84 – -1.11	**<0.001**
roi [Precentral_R] : GMvolume	1.49	1.24 – 1.73	**<0.001**	-1.49	-1.73 – -1.26	**<0.001**
roi [Precuneus_L] : GMvolume	-0.05	-0.32 – 0.21	0.687	0.03	-0.23 – 0.28	0.846
roi [Precuneus_R] : GMvolume	0.37	0.14 – 0.60	**0.001**	-0.34	-0.56 – -0.12	**0.002**
roi [Putamen_L] : GMvolume	-0.93	-1.95 – 0.09	0.075	0.83	-0.14 – 1.81	0.095
roi [Putamen_R] : GMvolume	-0.31	-1.73 – 1.11	0.668	0.35	-1.00 – 1.71	0.610
roi [Rectus_L] : GMvolume	1.45	0.84 – 2.06	**<0.001**	-1.48	-2.06 – -0.90	**<0.001**
roi [Rectus_R] : GMvolume	1.21	0.63 – 1.79	**<0.001**	-1.22	-1.78 – -0.66	**<0.001**
roi [Rolandic_Oper_L] : GMvolume	1.37	1.13 – 1.62	**<0.001**	-1.38	-1.61 – -1.14	**<0.001**
roi [Rolandic_Oper_R] : GMvolume	3.02	2.49 – 3.56	**<0.001**	-3.03	-3.54 – -2.52	**<0.001**
roi [Supp_Motor_Area_L] : GMvolume	0.19	-0.73 – 1.12	0.684	-0.11	-0.99 – 0.77	0.807
roi [Supp_Motor_Area_R] : GMvolume	0.38	-0.41 – 1.18	0.344	-0.37	-1.13 – 0.39	0.337
roi [SupraMarginal_L] : GMvolume	1.95	1.09 – 2.81	**<0.001**	-1.89	-2.71 – -1.07	**<0.001**
roi [SupraMarginal_R] : GMvolume	-0.49	-0.77 – -0.20	**0.001**	0.41	0.14 – 0.68	**0.003**
roi [Temporal_Inf_L] : GMvolume	-0.07	-0.14 – -0.00	**0.039**	0.05	-0.01 – 0.11	0.121
roi [Temporal_Inf_R] : GMvolume	0.10	0.04 – 0.16	**0.001**	-0.12	-0.18 – -0.07	**<0.001**
roi [Temporal_Mid_L] : GMvolume	0.19	0.12 – 0.25	**<0.001**	-0.22	-0.28 – -0.16	**<0.001**
roi [Temporal_Mid_R] : GMvolume	0.32	0.23 – 0.40	**<0.001**	-0.36	-0.44 – -0.27	**<0.001**
roi [Temporal_Pole_Mid_L] : GMvolume	1.41	0.65 – 2.17	**<0.001**	-1.47	-2.19 – -0.74	**<0.001**

roi [Temporal_Pole_Mid_R] : GMvolume	1.60	0.89 – 2.31	**<0.001**	-1.64	-2.32 – -0.96	**<0.001**
roi [Temporal_Pole_Sup_L] : GMvolume	1.19	-0.01 – 2.39	0.051	-1.23	-2.37 – -0.09	**0.035**
roi [Temporal_Pole_Sup_R] : GMvolume	2.26	1.51 – 3.01	**<0.001**	-2.30	-3.02 – -1.59	**<0.001**
roi [Temporal_Sup_L] : GMvolume	0.09	-0.05 – 0.24	0.195	-0.18	-0.31 – -0.04	**0.011**
roi [Temporal_Sup_R] : GMvolume	-0.03	-0.12 – 0.05	0.443	-0.00	-0.08 – 0.08	0.942
roi [Thalamus_L] : GMvolume	1.71	0.76 – 2.67	**<0.001**	-1.52	-2.43 – -0.60	**0.001**
roi [Thalamus_R] : GMvolume	1.01	0.25 – 1.77	**0.009**	-0.95	-1.68 – -0.23	**0.010**
**Random Effects**						
σ^2	0.16			0.15		
τ_{00}	0.03 subjects			0.03 subjects		
N	65 subjects			65 subjects		
Marginal R^2 / Conditional R^2	0.809 / 0.839			0.825 / 0.853		

Note. Results of linear mixed effect model predicting average controllability based on the interaction between region and regional gray matter , and region and gray matter volume. Region index extracted from AAL2 atlas and the TIV were additional predictors. σ^2: random effect variance; τ_{00}: between-subject variance; CI: bootstrapped 95% confidence intervals. p-values were computed via Wald-statistics approximation (treating t as Wald z).

Table S3: details of the model in Figure 3

	**Average controllability**			**Modal controllability**		
Predictors	*std. Beta*	*standardized CI*	*p*	*std. Beta*	*standardized CI*	*p*
TIV	-0.01	-0.09 – 0.08	0.850	0.01	-0.07 – 0.09	0.815
NodalDegree	0.38	0.34 – 0.43	**<0.001**	-0.38	-0.42 – -0.33	**<0.001**
GMvolume	0.14	0.02 – 0.25	**0.021**	-0.14	-0.25 – -0.02	**0.018**
roi [lh-bankssts]	-0.25	-0.46 – -0.04	**0.021**	0.25	0.04 – 0.45	**0.020**
roi [lh-caudalanteriorcingulate]	-0.39	-0.61 – -0.17	**0.001**	0.40	0.18 – 0.62	**<0.001**
roi [lh-caudalmiddlefrontal]	0.15	-0.04 – 0.33	0.120	-0.11	-0.30 – 0.07	0.228
roi [lh-cuneus]	1.28	1.08 – 1.48	**<0.001**	-1.31	-1.51 – -1.11	**<0.001**
roi [lh-entorhinal]	-0.51	-0.73 – -0.30	**<0.001**	0.53	0.31 – 0.74	**<0.001**
roi [lh-fusiform]	0.65	0.45 – 0.86	**<0.001**	-0.69	-0.89 – -0.49	**<0.001**
roi [lh-inferiorparietal]	-0.58	-0.82 – -0.34	**<0.001**	0.59	0.35 – 0.83	**<0.001**
roi [lh-inferiortemporal]	0.54	0.32 – 0.75	**<0.001**	-0.58	-0.80 – -0.37	**<0.001**
roi [lh-isthmuscingulate]	-0.05	-0.25 – 0.16	0.646	0.04	-0.16 – 0.24	0.693
roi [lh-lateraloccipital]	0.03	-0.22 – 0.27	0.834	-0.03	-0.27 – 0.21	0.806
roi [lh-lateralorbitofrontal]	0.98	0.78 – 1.18	**<0.001**	-1.01	-1.21 – -0.81	**<0.001**
roi [lh-lingual]	0.57	0.39 – 0.76	**<0.001**	-0.59	-0.78 – -0.41	**<0.001**
roi [lh-medialorbitofrontal]	1.00	0.81 – 1.19	**<0.001**	-1.02	-1.20 – -0.83	**<0.001**
roi [lh-middletemporal]	0.17	-0.04 – 0.38	0.119	-0.20	-0.41 – 0.01	0.063
roi [lh-parahippocampal]	-0.07	-0.28 – 0.15	0.549	0.05	-0.16 – 0.26	0.656
roi [lh-paracentral]	0.91	0.71 – 1.10	**<0.001**	-0.85	-1.04 – -0.66	**<0.001**
roi [lh-parsopercularis]	-0.28	-0.46 – -0.09	**0.004**	0.31	0.13 – 0.49	**0.001**
roi [lh-parsorbitalis]	-0.21	-0.43 – 0.00	0.053	0.22	0.01 – 0.43	**0.042**
roi [lh-parstriangularis]	-0.41	-0.61 – -0.22	**<0.001**	0.42	0.23 – 0.61	**<0.001**
roi [lh-pericalcarine]	1.54	1.33 – 1.75	**<0.001**	-1.59	-1.80 – -1.39	**<0.001**
roi [lh-postcentral]	1.41	1.18 – 1.64	**<0.001**	-1.34	-1.56 – -1.12	**<0.001**

roi [lh-posteriorcingulate]	-0.57	-0.77 – -0.37	**<0.001**	0.58	0.39 – 0.78	**<0.001**
roi [lh-precentral]	1.09	0.74 – 1.45	**<0.001**	-1.03	-1.38 – -0.68	**<0.001**
roi [lh-precuneus]	0.64	0.41 – 0.86	**<0.001**	-0.68	-0.90 – -0.46	**<0.001**
roi [lh-rostralanteriorcingulate]	-0.33	-0.53 – -0.12	**0.002**	0.33	0.13 – 0.54	**0.001**
roi [lh-rostralmiddlefrontal]	-0.10	-0.39 – 0.18	0.476	0.12	-0.16 – 0.40	0.400
roi [lh-superiorfrontal]	-0.83	-1.32 – -0.34	**0.001**	0.86	0.38 – 1.35	**<0.001**
roi [lh-superiorparietal]	0.46	0.19 – 0.74	**0.001**	-0.46	-0.73 – -0.19	**0.001**
roi [lh-superiortemporal]	-0.35	-0.58 – -0.12	**0.003**	0.32	0.09 – 0.55	**0.005**
roi [lh-supramarginal]	0.02	-0.21 – 0.25	0.857	0.00	-0.22 – 0.23	0.974
roi [lh-frontalpole]	-0.40	-0.63 – -0.16	**0.001**	0.41	0.17 – 0.65	**0.001**
roi [lh-temporalpole]	-0.51	-0.73 – -0.29	**<0.001**	0.53	0.31 – 0.75	**<0.001**
roi [lh-transversetemporal]	-0.19	-0.42 – 0.04	0.112	0.19	-0.04 – 0.41	0.112
roi [lh-insula]	-0.64	-0.86 – -0.42	**<0.001**	0.68	0.46 – 0.90	**<0.001**
roi [Left-Thalamus-Proper]	-0.88	-1.06 – -0.69	**<0.001**	0.90	0.71 – 1.08	**<0.001**
roi [Left-Caudate]	-0.59	-0.79 – -0.40	**<0.001**	0.61	0.42 – 0.80	**<0.001**
roi [Left-Putamen]	-0.53	-0.73 – -0.34	**<0.001**	0.55	0.35 – 0.74	**<0.001**
roi [Left-Pallidum]	-0.56	-0.78 – -0.34	**<0.001**	0.58	0.36 – 0.79	**<0.001**
roi [Left-Hippocampus]	-0.26	-0.45 – -0.07	**0.008**	0.24	0.05 – 0.43	**0.012**
roi [Left-Amygdala]	-0.51	-0.74 – -0.28	**<0.001**	0.53	0.30 – 0.75	**<0.001**
roi [Left-Accumbens-area]	-0.40	-0.66 – -0.15	**0.002**	0.42	0.17 – 0.68	**0.001**
roi [Right-Thalamus-Proper]	-0.80	-0.99 – -0.62	**<0.001**	0.82	0.64 – 1.00	**<0.001**
roi [Right-Caudate]	-0.60	-0.79 – -0.40	**<0.001**	0.61	0.42 – 0.80	**<0.001**
roi [Right-Putamen]	-0.44	-0.63 – -0.24	**<0.001**	0.44	0.24 – 0.63	**<0.001**
roi [Right-Pallidum]	-0.58	-0.80 – -0.37	**<0.001**	0.60	0.38 – 0.81	**<0.001**
roi [Right-Hippocampus]	-0.19	-0.38 – 0.00	0.054	0.17	-0.02 – 0.36	0.082

roi [Right-Amygdala]	-0.56	-0.78 – -0.34	**<0.001**	0.58	0.36 – 0.80	**<0.001**
roi [Right-Accumbens-area]	-0.40	-0.66 – -0.14	**0.002**	0.42	0.16 – 0.67	**0.001**
roi [rh-bankssts]	-0.23	-0.45 – -0.02	**0.033**	0.23	0.02 – 0.44	**0.032**
roi [rh-caudalanteriorcingulate]	-0.27	-0.49 – -0.05	**0.016**	0.27	0.06 – 0.49	**0.013**
roi [rh-caudalmiddlefrontal]	-0.07	-0.26 – 0.11	0.454	0.08	-0.10 – 0.27	0.362
roi [rh-cuneus]	0.42	0.22 – 0.62	**<0.001**	-0.44	-0.64 – -0.24	**<0.001**
roi [rh-entorhinal]	-0.50	-0.72 – -0.28	**<0.001**	0.51	0.30 – 0.73	**<0.001**
roi [rh-fusiform]	0.94	0.74 – 1.14	**<0.001**	-0.99	-1.19 – -0.79	**<0.001**
roi [rh-inferiorparietal]	-0.38	-0.65 – -0.10	**0.007**	0.37	0.10 – 0.64	**0.007**
roi [rh-inferiortemporal]	0.52	0.31 – 0.73	**<0.001**	-0.56	-0.76 – -0.35	**<0.001**
roi [rh-isthmuscingulate]	0.10	-0.10 – 0.31	0.335	-0.11	-0.32 – 0.09	0.272
roi [rh-lateraloccipital]	-0.08	-0.32 – 0.17	0.543	0.07	-0.17 – 0.31	0.550
roi [rh-lateralorbitofrontal]	0.87	0.68 – 1.07	**<0.001**	-0.92	-1.11 – -0.73	**<0.001**
roi [rh-lingual]	0.23	0.05 – 0.42	**0.015**	-0.25	-0.43 – -0.07	**0.007**
roi [rh-medialorbitofrontal]	0.91	0.72 – 1.10	**<0.001**	-0.94	-1.13 – -0.76	**<0.001**
roi [rh-middletemporal]	-0.26	-0.49 – -0.04	**0.021**	0.25	0.03 – 0.47	**0.026**
roi [rh-parahippocampal]	-0.02	-0.23 – 0.20	0.883	-0.00	-0.21 – 0.21	0.977
roi [rh-paracentral]	0.62	0.43 – 0.81	**<0.001**	-0.59	-0.78 – -0.41	**<0.001**
roi [rh-parsopercularis]	-0.40	-0.59 – -0.21	**<0.001**	0.42	0.23 – 0.61	**<0.001**
roi [rh-parsorbitalis]	-0.11	-0.32 – 0.11	0.328	0.11	-0.10 – 0.32	0.304
roi [rh-parstriangularis]	-0.56	-0.75 – -0.37	**<0.001**	0.57	0.38 – 0.76	**<0.001**
roi [rh-pericalcarine]	0.68	0.47 – 0.89	**<0.001**	-0.72	-0.93 – -0.52	**<0.001**
roi [rh-postcentral]	1.04	0.82 – 1.26	**<0.001**	-1.02	-1.23 – -0.81	**<0.001**
roi [rh-posteriorcingulate]	-0.53	-0.73 – -0.33	**<0.001**	0.54	0.35 – 0.74	**<0.001**
roi [rh-precentral]	0.79	0.50 – 1.08	**<0.001**	-0.78	-1.07 – -0.49	**<0.001**

roi [rh-precuneus]	0.88	0.66 – 1.09	**<0.001**	-0.92	-1.14 – -0.71	**<0.001**
roi [rh-rostralanteriorcingulate]	-0.42	-0.63 – -0.21	**<0.001**	0.43	0.22 – 0.64	**<0.001**
roi [rh-rostralmiddlefrontal]	-0.66	-0.95 – -0.37	**<0.001**	0.65	0.37 – 0.94	**<0.001**
roi [rh-superiorfrontal]	-0.80	-1.27 – -0.34	**0.001**	0.80	0.33 – 1.26	**0.001**
roi [rh-superiorparietal]	0.65	0.39 – 0.91	**<0.001**	-0.66	-0.92 – -0.40	**<0.001**
roi [rh-superiortemporal]	-0.64	-0.87 – -0.42	**<0.001**	0.63	0.41 – 0.85	**<0.001**
roi [rh-supramarginal]	0.12	-0.09 – 0.34	0.261	-0.12	-0.33 – 0.09	0.267
roi [rh-frontalpole]	-0.24	-0.48 – -0.00	**0.047**	0.25	0.01 – 0.48	**0.038**
roi [rh-temporalpole]	-0.49	-0.71 – -0.28	**<0.001**	0.51	0.30 – 0.73	**<0.001**
roi [rh-transversetemporal]	-0.31	-0.55 – -0.07	**0.011**	0.32	0.08 – 0.55	**0.009**
roi [rh-insula]	-0.68	-0.89 – -0.47	**<0.001**	0.68	0.47 – 0.88	**<0.001**
sex [male]	-0.04	-0.22 – 0.13	0.625	0.04	-0.13 – 0.21	0.650
NodalDegree * GMvolume	0.08	0.04 – 0.12	**<0.001**	-0.08	-0.12 – -0.04	**<0.001**
**Random Effects**						
σ^2	0.30			0.30		
τ_{00}	0.05 $_{subjects}$			0.05 $_{subjects}$		
N	48 $_{subjects}$			48 $_{subjects}$		
Observations	3779			3779		

Note. Results of linear mixed effect model predicting average controllability based on the interaction between nodal degree and regional gray matter volume. Region index and the TIV were additional predictors. σ^2 : random effect variance; r_{00}: between-subject variance; CI: bootstrapped 95% confidence intervals. p-values were computed via Wald-statistics approximation (treating t as Wald z) and Bonferroni corrected.
